# Synthetic Cathinones' Comprehensive Screening and Classification by Voltammetric and Chemometric Analyses: A Powerful Method for On-Site Forensic Applications

**DOI:** 10.1021/acs.analchem.4c04059

**Published:** 2024-10-25

**Authors:** Camila Diana Lima, Luciano C. Arantes, Lara L. Machado, Thiago R. L. C. Paixão, Wallans T. P. dos Santos

**Affiliations:** † Departamento de Química, Universidade Federal dos Vales do Jequitinhonha e Mucuri, Diamantina, Minas Gerais 39100-000, Brazil; ‡ Departamento de Farmácia, Universidade Federal dos Vales do Jequitinhonha e Mucuri, Diamantina, Minas Gerais 39100-000, Brazil; § Laboratório de Química e Física Forense, Instituto de Criminalística, Polícia Civil do Distrito Federal, Brasília, Distrito Federal 70610-907, Brazil; ∥ Departamento de Química Fundamental, Instituto de Química, Universidade de São Paulo, São Paulo, São Paulo 05508-000, Brazil

## Abstract

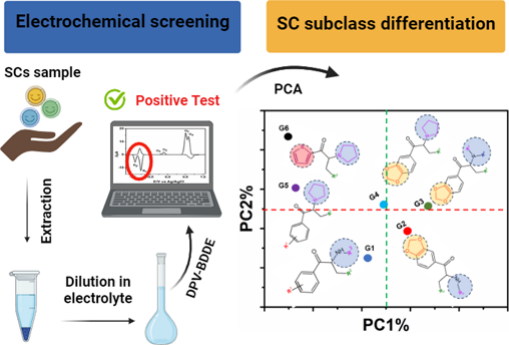

The use of synthetic cathinones (SCs) has increased in recent years, posing significant public health problems due to their adverse effects and potential for fatal poisonings. The structural diversity and rapid emergence of new SC analogues create challenges for law enforcement and drug screening techniques. This work presents for the first time the electrochemical detection of SCs using differential pulse voltammetry (DPV) on a boron-doped diamond electrode (BDDE). We analyzed 15 SCs, including well-known compounds such as mephedrone, methylone, and ephylone, revealing distinct electrochemical profiles with two characteristic reduction peaks (R_1_ and R_2_). The method was optimized in Britton–Robinson buffer (0.1 mol L^–1^, pH 8.0) and demonstrated a high selectivity and sensitivity. Multivariate statistical methods, including principal component analysis and hierarchical cluster analysis, classified SCs into six distinct groups. The DPV optimization and analytical parameter determination, including the limit of detection (LOD), were performed for the least electroactive SC, 4′-methyl-α-pyrrolidinohexanophenone, yielding an LOD of 3.8 μmol L^–1^, suitable for screening street samples. Interference studies with common illicit drugs and adulterants confirmed the selectivity of the DPV-BDDE method. Preliminary identification of SCs in 46 real seized samples was successfully performed using this method with results validated by liquid chromatography–mass spectrometry (LC–MS). The method also identified three SCs not included in the original set: bupropion, benzylone, and dipentylone. The DPV-BDDE method offers a rapid, robust, and portable approach for the selective screening of SCs in forensic applications, demonstrating significant advantages over traditional colorimetric tests.

## Introduction

The restriction and criminalization of psychoactive substances in recent years have driven an unprecedented growth in the number, type, and availability of numerous synthetic substances, known worldwide as new psychoactive substances (NPS).^1^ NPS quickly became popular as drugs of abuse due to their questionable legal status, lower cost, ease of acquisition, and similar or more potent effects compared to traditional proscribed drugs such as cannabis, cocaine, heroin, LSD, MDMA (“ecstasy”), and methamphetamine.^2,3^ According to the United Nations Office on Drugs and Crime (UNODC), 1241 NPS have been reported to date, with stimulant substances such as synthetic cathinones (SCs) being the second most reported class, accounting for 206 substances or 18.07% of the reported NPS.^4^

**Figure 1:**
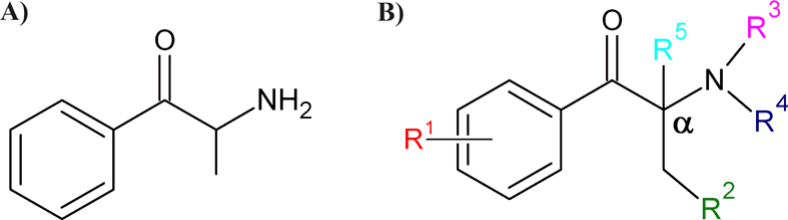
(A) Chemical structure of cathinone. (B) Prototype structure of SCs showing key functionalization sites.

SCs are designer drugs that generate β-ketogenic analogues of phenethylamines.^5^ Their chemical structures are related to classical amphetamine-type stimulants (amphetamine and methamphetamine) and MDMA.^6,7^ SCs are chemically engineered based on the natural psychoactive alkaloid cathinone (Figure 1A), found in the leaves of the plant khat (*Catha edulis*).^8−10^ The structural prototype of SCs (Figure 1B) is used by clandestine laboratories to guide the synthesis of new derivatives. By introducing small chemical modifications at specific locations such as the aromatic ring (R^1^), alkyl chain (R^2^ and R^5^), and amine group (R^3^ and R^4^), a variety of SCs analogues can be synthesized.^7^ Depending on the type of substituent introduced, SCs can be classified into four subfamilies: *N*-alkylated, *N*-pyrrolidine, 3,4-methylenedioxy-*N*-alkyl, and 3,4-methylenedioxy-*N*-pyrrolidine.^7^

The popularity of SCs, combined with their toxicity and adverse effects, and the rapid production of new analogues by clandestine laboratories pose significant public health and security challenges.^11,12^ The number of SCs reported to UNODC increases annually, creating operational challenges due to the lack of certified analytical standards for proper identification, reliable reference data, and effective screening tests.^4,7,13^ At least 22 countries control groups of substances through generic legislation, anticipating the control of new derivatives from defined chemical structures, such as the cathinone core. The Brazilian Health Regulatory Agency (ANVISA) has controlled the SC class since 2017.^14^

Preliminary determination of illicit substances in seized drug samples is crucial for forensic investigations and law enforcement, providing essential information and serving as immediate evidence for issuing arrest reports and tracing criminal networks. For screening SCs in seized samples, UNODC recommends the use of the Zimmermann or Janovsky colorimetric tests as the most appropriate presumptive methods.^15^ The former relies on a nucleophilic aromatic substitution reaction between Zimmermann’s reagent and certain SC derivatives (Figure S1), producing a color change that suggests the presence of SCs.^16^

Although practical, the Zimmermann test has limitations. It is not effective in detecting all SCs, presenting positive results only for derivatives with specific structural features, such as hydrogen in R^5^ plus R^3^ and R^4^ with nonbulky substituents (Figure 1B). Furthermore, colorimetric methods have several drawbacks including low discrimination power and cross-reactivity with substances structurally similar to the target analyte, which can produce false-positive results.^17,18^ Additionally, the purposeful addition of dyes and adulterants can further complicate result interpretation.^18,19^

In the search for portable and efficient presumptive tests to assist police and harm reduction efforts in detecting and identifying illicit drugs on-site, alternative methods have been developed based on techniques such as attenuated total reflectance Fourier transform infrared spectroscopy (ATR-FTIR), Raman spectroscopy, and near-infrared (NIR) spectroscopy.^20^ However, these techniques have limitations when applied to samples with complex matrices, as they cannot be satisfactorily analyzed by simple spectral matching alone. Additionally, Raman spectroscopy can suffer from fluorescence interference with white and colored samples.^21^ On the other hand, electrochemical methods stand out as viable analytical tools due to their speed, cost-effectiveness, portability, sensitivity, and ease of operation.^22^ Several electrochemical sensors have been developed for screening NPS in seized drugs,^25−31^ including some SCs.^23−25^ While these sensors have been effective, the boron-doped diamond electrode (BDDE) offers some additional advantages for detecting drugs in forensic samples^23,27,32−34^ due to its high stability, low background current, and wide potential window.^35^ Additionally, as an unmodified sensor, the BDDE allows simpler and faster electroanalysis for SC detection compared with other reported sensors.

Chemometric approaches have recently been combined with electrochemical methods to reduce subjectivity in the analyst’s decision-making process.^36,37^ To date, only two studies^38,39^ have addressed chemometric processing of electrochemical data from SCs for drug sample recognition. Shishkanova et al.^38^ and Dragan et al.^39^ demonstrated the promising use of electrochemical techniques with chemometrics for SC identification and control. However, none of these studies explored the structural diversity or electrochemical profiles of the four SC families.

We present, for the first time, the electrochemical behavior and detection of 15 SCs using differential pulse voltammetry (DPV) and BDDE. We also introduce an innovative approach by applying chemometric treatments to DPV data to distinguish SC electrochemical profiles and relate them to their chemical structures. Overall, the proposed method offers a selective and generic screening test for SC groups in forensic analysis, providing a comprehensive solution for preliminary identification across the entire SC class.

## Experimental Section

### Chemicals and Samples

All solutions were prepared with deionized water with a resistivity of at least 18.2 MΩ cm (at 25 °C) obtained from a Milli-Q system (Millipore, USA). Analytical standards of SCs, including mephedrone (4-MMC), ethcathinone, 4-methyl-pentedrone (4-MPD), methylone (bk-MDMA), ethylone (bk-MDEA), eutylone (bk-EBDB), *N*-ethyl-pentylone (ephylone), 3,4-methylenedioxy-*N*-tert-butylcathinone (MDPT), dibutylone (bk-DMBDB), 3,4-methylenedioxy-pyrovalerone (MDPV), 3,4-methylenedioxy-α-pyrrolidinohexanophenone (MDPHP), 4′-methyl-α-pyrrolidinohexanophenone (MPHP), α-pyrrolidinopentiophenone (α-PVP), 3′,4′-tetramethylene-α-pyrrolidinovalerophenone (TH-PVP), and α-pyrrolidinopentiothiophenone (α-PVT) (Table S1), were obtained from Cayman Chemical Company (Ann Arbor, MI, USA) in powder form and solubilized in methanol to obtain a 1.0 × 10^–2^ mol L^–1^ stock solution. The stock solution was diluted in a supporting electrolyte for electrochemical measurements. The electrochemical behavior of the 15 cathinone analogues was studied in a Britton–Robinson (BR) buffer solution prepared from a mixture of boric, phosphoric, and acetic acids at different pH values (from 2.0 to 12.0). For SC detection by the proposed method, the following compounds were evaluated as possible interferences: cocaine (COC), caffeine (CAF), paracetamol (PAR), 3,4-methylenedioxymethamphetamine (MDMA), 3,4-methylenedioxyethylamphetamine (MDEA), methamphetamine (MA), amphetamine (A), ketamine (KET), procaine (PROC), benzocaine (BENZ), lidocaine (LID), 3-chlorophenylpiperazine (mCPP), and 1-benzylpiperazine (BZP). All analytical standards were purchased from Cayman Chemical Company (Ann Arbor, MI, USA).

Samples of seized tablets (*N* = 46) were provided by the Civil Police of the Federal District (PCDF), Brazil, where the presence of SCs analogues and other interferents was previously confirmed by liquid chromatography quadrupole time-of-flight mass spectrometry analysis (LC-Q-TOF-MS). The samples were obtained after an extraction stage carried out at the Institute of Criminalistics of the Civil Police of the Federal District (IC/PCDF) and the Civil Police of the State of Minas Gerais in Brazil. First, 100 mg of each sample was ground, homogenized, diluted in 1 mL of methanol, and sonicated for 10 min. Subsequently, this extract was diluted 800-fold in an electrolyte that supports the detection of SCs by the proposed electrochemical method.

### Instruments and Apparatus

Electrochemical measurements were performed using an μAutolab III potentiostat/galvanostat (Metrohm Autolab, Utrecht, The Netherlands) controlled by NOVA version 2.1 software. A BDD film (geometric area of 0.13 cm^2^) on a silicon wafer (8000 ppm of doping, acquired from NeoCoat SA, La Chaux-de-Fonds, Switzerland), a platinum wire, and a miniaturized Ag/AgCl (KCl sat.) sensor were used as the working, counter, and reference electrodes, respectively. The BDDE surface was cathodically treated by applying a current of +1.0 mA for 30 s followed by −30 mA for 90 s in 0.5 mol L^–1^ H_2_SO_4_.^40^ Cyclic voltammetry (CV) and DPV techniques were used for profiling, electrochemical studies, and detection of 15 SC analogues. The analyses by LC-Q-TOF-MS were carried out at the IC/PCDF using a 1290 Infinity ultra-high-performance liquid chromatography system coupled to a 6540 quadrupole time-of-flight mass spectrometer (Agilent Technologies, Santa Clara, CA, USA). A Dual Agilent Jet Stream Electrospray Ionization (Dual AJS ESI) interface was used to transfer analytes from the LC to the MS. Accurate mass compounds were detected and reported using Agilent MassHunter Qualitative Analysis software version B 06.00 and Personal Compound Database and Library version B 02.00 (PCDL).

### Electrochemical Measurements

Initial electrochemical studies of the 15 SCs were carried out by DPV at pH values ranging from 2.0 to 12.0. The screening and detection method for the 15 SCs was optimized using DPV with BDDE under optimal parameters: amplitude of 80 mV, step potential of 10 mV, modulation time of 50 ms, and time interval of 0.1 s, obtained for the model molecule MPHP. Before each measurement, a cathodic treatment was performed. Subsequently, the BDDE was electrochemically conditioned using CV in a BR buffer solution of 0.1 mol L^–1^ at pH 8.0 for 15 cycles between −1.50 and +2.2 V (vs Ag/AgCl) at a scan rate of 800 mV s ^–1^. Electrochemical measurements in BDDE were performed in anodic (−2.0 to +2.0 V) and cathodic (+2.0 to −2.0 V) sweeps using 100 μL of standard solution or sample to cover the entire electrode. Voltammograms obtained by DPV were subjected to background subtraction using a polynomial fit with Origin software (OriginPro 2016, Northampton, MA). The limits of detection (LOD) and quantification (LOQ) were evaluated using the analytical curve parameters according to the equations LOD = 3.3σ/*S* and LOQ = 10σ/*S*, where σ is the standard deviation of the response and *S* is the slope of the calibration curve.^41,42^

### Chemometrics

Principal component analysis (PCA) and hierarchical cluster analysis (HCA) were performed using Statistica 13.5 (StatSoft Inc., USA). The analysis was carried out using current and potential values from the DPVs. Different preprocessing methodologies were applied to the raw voltammograms, such as cutting in the potential region between −1.60 to +1.60 V and +1.80 to −1.80 V for anodic and cathodic sweeps, respectively, voltammogram baseline correction by background subtraction using a polynomial fit with Origin software (OriginPro 2016, Northampton, MA), and normalization of current values. All DPVs were recorded three times for each SC pattern in random order, with cathodic treatment of the BDDE surface performed between measurements.

## Results and Discussion

### Electrochemical Behavior of SCs on BDDE

Initial DPV studies of 15 SCs in BR buffer (0.1 mol L^–1^, pH 2.0–12.0) using BDDE showed that all molecules are electroactive (Figures S2B–S7B and S2C–S7C). The redox processes for all SCs were pH-dependent, with peak potentials (*E*_p_) shifting to more negative values as pH increased (Figures S3B−S7B for anodic peaks and Figures S2C−S7C for cathodic peaks). This behavior may be related to the p*K*_a_ distribution of the SCs, where values between 7.0 and 8.5 were observed, and the pH distribution from 0.0 to 14.0, in which all analytes studied presented two species: a cationic form in nitrogen, predominant at pH < p*K*_a_, and a deprotonated (neutral) form at pH > p*K*_a_.^43^ In addition, all SCs from pH 6.0 showed two reduction peaks (R_1_ and R_2_) in BDDE (Figures S2C–S7C). The BDDE offers a wider potential window than screen-printed carbon electrodes (SPCEs) and glassy carbon electrodes (GCEs), enabling observation of all SC electrochemical processes with greater stability and easier reusability.

To the best of the authors’ knowledge, the R_2_ cathodic process (∼−1.60 V vs Ag/AgCl) has never been reported as a characteristic peak for the SC class in any electrochemical sensor. A process at ∼−1.45 V (vs Ag/AgCl) in BR buffer pH 8.0 was reported as the second reduction for ephylone in a lab-made, chemically deposited boron-doped diamond electrochemical sensor (LM-SP/BDDE).^44^ However, this process was not observed in other SCs analyzed under the same conditions in the same paper (MDPHP, α-PVP, ethylone, and mephedrone), suggesting it may have been misinterpreted as the second reduction due to a limited potential window.^44^

The R_1_ and R_2_ peaks can be considered as a fingerprint for the class of SCs, as no cathodic processes have been identified in the same region of *E*_p_ in other drugs that have a phenylethylamine nucleus, such as NBOMes, NBOH,^45,46^ amphetamines, methylenedioxyamphetamine (MDA), MDMA,^47^ and MDEA.^48^ This work presents new electrochemistry signature for the class of SC analogues, allowing them to be differentiated from other synthetic drugs. To guide the choice of the working pH of the SC screening method proposed in this work, PCA (Figure S8) was used to treat the DPVs of the pH study for all analytes in the anodic scan (Figures S2B–S7B).

As plotted in Figure S8A, the score plot of the first two principal components (PC1 and PC2) showed that at pH 8.0 of 0.1 mol L^–1^ BR buffer, 73% of the potential and actual data variance was explained. The result presented in the 3D scatterplot (Figure S8B) shows a distribution of SCs in the four quadrants, enabling effective distinction of *E*_p_ between the investigated analytes. Furthermore, according to the DPVs in Figures S2B–S7B and S2C–S7C, pH 8.0 allowed the visualization of the greatest number of redox processes in SCs, and the R_1_ and R_2_ processes presented the highest current signal.

Complementary CV studies were performed for all tested SCs, showing that these drugs exhibit irreversible redox processes in BDDE (Figures S2A–S7A). Furthermore, the control of mass transport of SCs on the surface of BDDE was evaluated by CV at different scan rates (*v*) in 0.1 mol L^–1^ BR buffer (pH 8.0) using the reduction process (R_1_), as shown in Figures S9 and S10. This scan rate study indicates that the electrochemical process of SCs is diffusion-controlled in BDDE, except for TH-PVP and MDPHP, which showed α > 0.5, indicating mixed mass transport control. Furthermore, untreated, cathodic, and anodic pretreatment studies on the BDDE surface were performed for SC detection using MPHP as the model molecule (Figure S11). As shown in Figure S11 and discussed in the SI, the cathodic pretreatment of the BDDE surface was chosen for the application of the proposed method, as the R_2_ cathodic process for SCs is only observed with this procedure.

### Differentiation of Subclasses of SCs by Unsupervised Chemometric Methods

**Figure 2:**
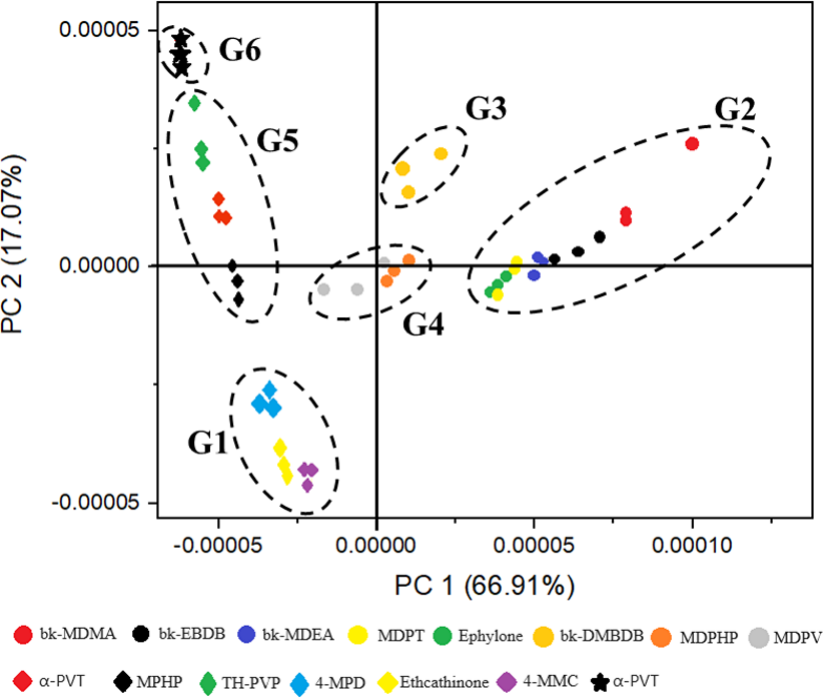
PCA score plot of 15 SC standards, detailed by DPV in BR buffer (0.1 mol L^–1^, pH 8.0).

To classify the SCs studied, unsupervised chemometric analyses—PCA (Figure 2) and HCA (Figure S12)—were applied to the DPV data obtained under optimized conditions, exploring both anodic and cathodic scans. The score plot of 15 SC standards (Figure 2) shows that PC1 and PC2 explain 66.91 and 17.07% of the variance, respectively. PCA revealed the separation of six SC groups based on the voltammetric profile, determined by the substituents at R^1^, R^3^, and R^4^ (Figure 1B). HCA (Figure S12) complemented PCA by quantitatively assessing sample similarity, confirming the six groups classified by PCA (Figure 2).

**Figure 3:**
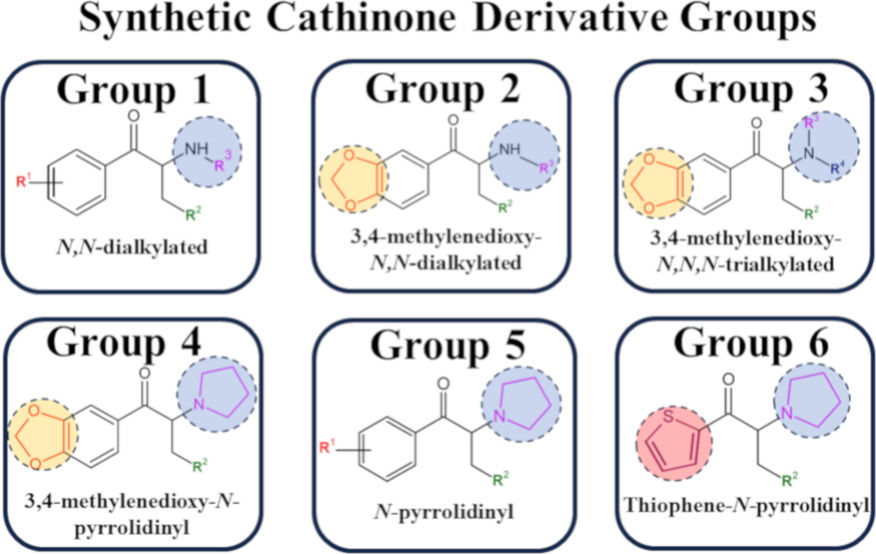
Proposed classification scheme for SCs based on their electrochemical profiles, influenced by substitutions at R^1^, R^3^, R^4^, and the presence of a thiophene ring.

Based on the results of chemometric analyses, a new generic classification for SCs was proposed, grounded in their electrochemical profiles, nitrogen substitution in the alkyl chain, the presence or absence of the 3,4-methylenedioxy substituent at R^1^, and aromatic ring replacement by the thiophene ring. The new groups are presented in Figure 3.

Group 1 (G1) includes *N*-alkylated SCs with a disubstituted amine in the alkyl chain (e.g., mephedrone, ethcathinone, and 4-MPD (Figure 3). Mephedrone and 4-MPD presented a DPV profile in the anodic scan with a cathodic process (R_1_) at approximately −1.40 V and an anodic process (O_1_) at +1.19 and +1.10 V (vs Ag/AgCl), respectively (Figure S13A). For ethcathinone (Figure S13A), only the R_1_ process was visualized in the DPV anodic scan (∼−1.40 V vs Ag/AgCl). The oxidation peak of the O_1_ was observed at ∼+1.27 V only in CV (Figure S2A), as the O_1_ process forms a poorly defined peak at scan rates above 50 mV s^–1^. In DPV cathodic scanning (Figure S13A), two cathodic processes (R_1_ and R_2_) were visualized at ∼−1.40 and ∼−1.60 V (vs Ag/AgCl) for G1 SCs.

Groups 2, 3, and 4 (G2–G4, Figure 3) are characterized by a 3,4-methylenedioxy ring substitution at R^1^ (Figure 1B) with varying degrees of nitrogen substitution, resulting in varying DPV profiles and the number of anodic processes. Group 2 (methylone, ethylone, eutylone, ephylone, and MDPT) consists of *N*-alkylated SCs with a disubstituted nitrogen.

Electrochemical studies of G2 SCs (Figure S13B,C) showed a DPV profile in the anodic sweep direction with a reduction peak at ∼−1.40 V (R_1_) and three anodic processes at ∼+0.60 V (O_1_), +1.0 V (O_2_), and +1.25 V (O_3_) (vs Ag/AgCl). Furthermore, the DPV cathodic scan for these SCs showed an oxidation peak at ∼+1.30 V for O_3_ and two cathodic processes, R_1_ and R_2_, at ∼−1.40 and ∼−1.60 V (vs Ag/AgCl), respectively.

Group 3, represented by dibutylone, differs from G2 structures by having a trisubstituted nitrogen in its side chain. The replacement by alkyl groups in positions R^3^ and R^4^ caused the DPV voltammogram (Figure S14A) of dibutylone in the anodic scan to present four anodic processes at ∼+0.60 V (O_1_), +0.83 V (O_2_), +1.03 V (O_3_), and +1.19 V (O_4_) and one cathodic process (R_1_) at ∼−1.40 V (vs Ag/AgCl). In the DPV cathodic scan, two anodic processes were observed at +1.30 and +1.0 V, corresponding to O_4_ and O_3_, respectively, and two cathodic processes, R_1_ (∼−1.30 V) and R_2_ (∼−1.60 V) (vs Ag/AgCl).

For group 4 (G4), the replacement of the side chain of MDPV and MDPHP with an *N*-pyrrolidine ring produced DPV voltammograms (Figure S14B) in the anodic sweep with five anodic peaks at ∼+0. 60 V (O_1_), +0.78 V (O_2_), +0.90 V (O_3_), +1.01 V (O_4_), and +1.19 V (O_5_) and a cathodic peak (R_1_) at ∼−1.40 V (vs Ag/AgCl). In the DPV cathodic scan, MDPV and MDPHP showed two anodic processes at ∼+1.30 and +1.0 V (vs Ag/AgCl), corresponding to O_5_ and O_4_, respectively, and two cathodic processes, R_1_ (∼−1.30 V) and R_2_ (∼−1.60 V) (vs Ag/AgCl).

Group 5 (G5) SCs (MPHP, α-PVP, and TH-PVP) (Figure 3) are characterized by a trisubstituted nitrogen in the alkyl side chain, forming a pyrrolidine ring. These SCs exhibited DPV voltammograms in the anodic scan (Figure S14C) with a reduction peak (R_1_) at ∼−1.30 V and two anodic peaks near +0.90 V (O_1_) and +1.0 V (O_2_) (vs Ag/AgCl). In the DPV cathodic scan, an anodic process was identified at ∼+1.0 V (O_2_) (vs Ag/AgCl) and two cathodic processes, R_1_ (∼−1.30 V) and R_2_ (∼−1.60 V) (vs Ag/AgCl).

Among the SCs studied, α-PVT is unique with a thiophene ring (Figure 3) instead of an aromatic ring common to the other cathinones. Despite a similarity of almost 60% to G5 (Figure S12), α-PVT is classified as group 6 (G6) due to its distinct electrochemical profile. According to the DPV shown in Figure S14D, α-PVT exhibited in the anodic scan one reduction process at ∼−1.3 V and four anodic processes close to −0.31 V (O_1_), −0.17 V (O_2_), +0.82 V (O_3_), and +0.95 V (O_4_) (vs Ag/AgCl). In the cathodic scan, two oxidation peaks at ∼+1.0 V (O_3_) and ∼+1.3 V (O_4_) and two reduction peaks at less cathodic potentials than the other SCs, R_1_ (−1.22 V) and R_2_ (−1.47 V) (vs Ag/AgCl), were identified in the DPV. Based on the work of Schram et al.^26^ and Pedersen et al.,^49^ mechanistic proposals are included and discussed in the SI for redox processes observed in the CS groups (G1–G6) (Figures S15–S18).

There have been reports in the literature of SCs exhibiting different electrochemical profiles depending on the substituents (R^1^, R^2^, R^3^, and R^4^) in the basic structure of natural cathinone.^26,27,31^ However, none of these studies systematically evaluated the voltammetric profiles of a large number of SCs with diverse structures. This study assigns redox processes to the different substituents in the basic structure of cathinone, providing a specific class signal that allows for generic classification. This systematic approach to classifying SCs based on their electrochemical profiles is demonstrated in this work.

### Detection of SCs by the DPV Technique

To obtain the best conditions for detecting SCs, the instrumental parameters of the DPV technique were optimized by univariate tests and 100 μmol L ^–1^ SC model (MPHP) with the lowest redox signal in BR buffer (0.1 mol L^–1^ pH 8.0). Under optimized conditions, the stability of electrochemical responses for SC detection using BDDE with the DPV technique was evaluated for intraday and interday repeatability (*N* = 5) of 100 μmol L^–1^ for all SC standards (Table S2). The DPV and square-wave voltammetry (SWV) techniques were compared for the detection of SCs using MPHP as the model analyte (Figure S19). As presented and discussed in the Supporting Information, DPV demonstrated higher stability of electrochemical responses than SWV.

The R_1_ process of all cathinones studied presented relative standard deviations (RSDs) lower than 13.0% for *I*_p_ and 1.0% for *E*_p_, in line with analytical recommendations (<20%) (Table S2).^41,42^ Additionally, a calibration curve was established in BR buffer (0.1 mol L ^–1^, pH 8.0) using increasing concentrations of the SC model (MPHP) from 1 to 100 μmol L ^–1^ (Figure S19A,B) to determine the LOQ and LOD for the proposed method. A linear concentration range between 15 and 100.0 μmol L^–1^ (*r*^2^ > 0.99), an LOQ of 11.5 μmol L^–1^, and an LOD of 3.8 μmol L^–1^ were obtained for the MPHP R_1_ process. The LOQ and LOD values are sufficiently low for detecting and quantifying SCs in seized forensic samples.

### Interference Studies and Application in Real Forensic Samples

Seized SC samples are often found in association with adulterants, either to enhance psychotropic effects or to reduce production costs. To verify the selectivity of the proposed SC screening method, an interference study was conducted. Caffeine, paracetamol, anesthetic medications, and other traditional illicit drugs such as amphetamine, methamphetamine, MDMA, MDEA, BZP, mCPP, and cocaine were tested as potential interferents. Figures S20 and 4 show the DPV scans recorded on BDDE, both anodic (A) and cathodic (B) scans, for the interferents and the electrochemical profiles obtained for the six SC groups proposed in this work.

**Figure 4:**
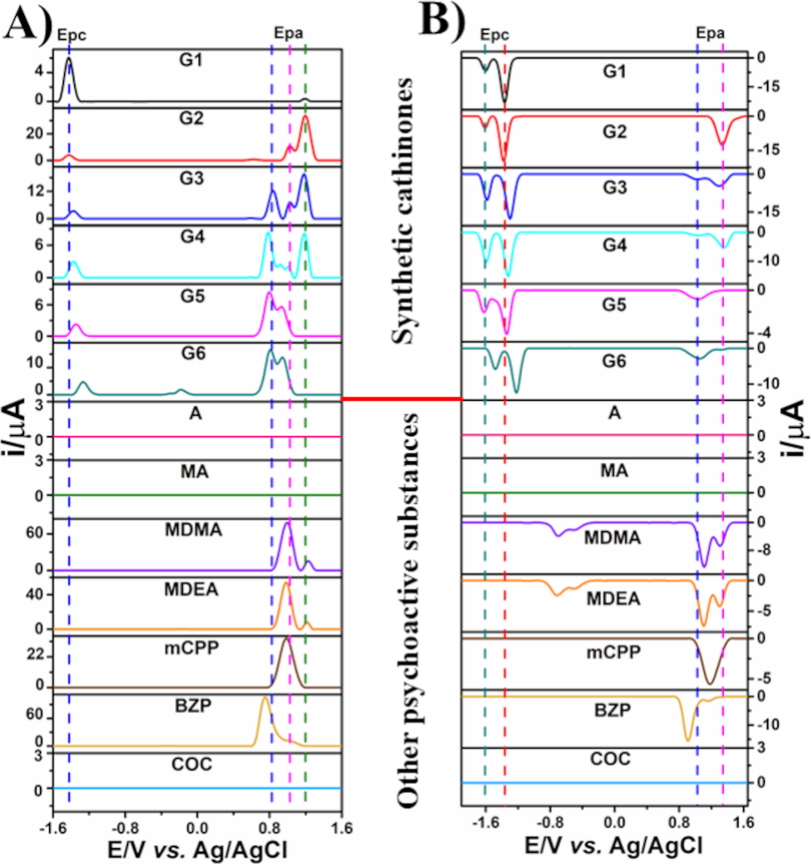
DPVs on BDDE using anodic (A) and cathodic (B) scans for SC representatives from groups G1–G6 and other traditional illicit drugs.

Figure S20A shows that the interferents produced some oxidation processes in BDDE, such as CAF at +1.30 V, PAR at +0.21 V, BENZ at +0.77 V, LID at +0.87 V, PROC at +0.77 V, and KET at +1.09 V (vs Ag/AgCl). The oxidation processes of BENZ, LID, PROC, and KET are relatively close to those of the SCs of the six groups (G1–G6). However, even in the presence of these interferents, preliminary identification of cathinones by DPV by anodic scanning is not compromised. This is because all SC derivatives from the six groups show a cathodic process between −1.4 and −1.2 V (vs Ag/AgCl). For a more accurate identification of SCs in the presence of BENZ, LID, PROC, KET, CAF, and PAR, combined DPV from both anodic and cathodic scanning directions can be used. In the cathodic scan (Figure S20B), the two characteristic cathodic processes of SCs are more visible and exhibit a higher *I*_p_. Notably, although PAR showed a reduction process in BDDE with *E*_p_ at −0.24 V (vs Ag/AgCl), this process does not overlap with the reductions (R_1_ and R_2_) used to detect the SC derivatives from the six groups.

Amphetamine, methamphetamine, and cocaine showed no electrochemical response (Figure 4), indicating that DPV can selectively detect SC derivatives from groups G1, G2, G3, G4, G5, and G6 in seized samples containing these illicit substances. Other tested illicit drugs produced oxidation processes in BDDE, such as MDMA and MDEA at +1.00 and +1.20 V, mCPP at +0.99 V, and BZP at +0.76 and +1.00 V (vs Ag/AgCl). The oxidation processes of these drugs could partially or completely overlap with at least one of the oxidations of the six SC groups in BDDE. However, the overlap of the oxidation peaks between the interferents and SCs in the anodic scan does not hinder the preliminary identification of cathinones (Figure 4A), as they can still be identified by the presence of the R_1_ cathodic process.

It is also important to note that the proposed method is effective for screening samples containing MDMA and MDEA, as these substances exhibit reduction processes around −0.7 and −0.5 V (vs Ag/AgCl) in BDDE, which do not overlap with the R_1_ and R_2_ reductions of the SC derivatives (Figure 4B). BZP and mCPP exhibited electrochemical responses similar to those of MDMA and MDEA, but they did not interfere with SC detection. Therefore, as shown in Figure 4, using DPV detection with both anodic and cathodic scanning, it is possible to selectively identify SCs in seized samples containing amphetamine-type stimulants, cocaine, and piperazine class drugs such as BZP and mCPP.

### Method Application for Detection of SCs in Real Samples

To demonstrate the potential application of the proposed method in real forensic scenarios, electrochemical screening of 46 seized samples was performed after ultrasound-assisted extraction in 1.0 mL of methanol, followed by an 800-fold dilution in the electrolyte support. Figure S21 shows the DPVs of the 46 seized samples in combined anodic and cathodic scanning using BDDE with baseline correction, while Figure S22 shows an example of DPV data without baseline correction for sample 4.

All samples with DPV showing the cathodic processes R_1_ (∼−1.30 V) and R_2_ (∼−1.60 V) (vs Ag/AgCl) were considered positive for SCs by the proposed method (Figure S21). Samples showing only the cathodic process R_1_ (∼−1.30 V vs Ag/AgCl) were also considered positive, as the R_2_ process has lower sensitivity. Samples 7, 26, 27, 30, 32, and 40 are examples of this behavior. Samples that showed neither R_1_ nor R_2_ were considered negative.

Although the main objective of this work is the screening of SCs in forensic samples, it is worth highlighting that the DPV-BBDE method also detected interferents, such as caffeine and MDMA/MDEA in 32.6% of the tested samples. Caffeine-containing samples showed one process at +1.30 V (samples 3, 19, 20, 23, 25, 29, 30, 33, 34, 35, 36, 38, 39, and 41), while MDMA/MDEA-containing samples exhibited two processes at −0.70 and −050 V (samples 7 and 46) (vs Ag/AgCl). The screening results obtained by the DPV-BDDE method were compared to the analysis data obtained by using the LC–MS chromatographic method (Table S3).

Of the 46 samples analyzed by the proposed method, 42 were positive and four were negative for SCs, showing 100% agreement with the results obtained by the gold standard method (LC–MS) (Table S3). The DPV-BDDE method classified the positive samples according to the SC group to which the detected analyte belonged (Figure S21). Samples with a single anodic process (∼+1.2 V), three anodic processes (∼+0.6, ∼+1.0, and ∼+1.2 V), and four anodic processes (∼+0.6, ∼+0.8, ∼+1.0, and ∼+1.2 V) (vs Ag/AgCl) were classified as SCs of groups G1, G2, and G3, respectively. For samples containing mixtures of SCs (samples 8 and 28), the DPV-BDDE method assigned them to the cathinone derivative group with a higher number of anodic processes.

Notably, three SCs not included in the 15 SCs used to develop the electrochemical method were detected among the seized samples and correctly classified: bupropion, a controlled medication in Brazil used to support smoking cessation and as an antidepressant; 3,4-methylenedioxy-*N*-benzylcathinone (BMDP), an NPS also known as benzylone; and dipentylone (*N*-ethyl pentylone), recently included in Schedule II of the Convention on Psychotropic Substances of 1971^50^ (Tables S1 and S3). This demonstrates that the method is sufficiently robust and specific to detect SCs in complex matrices, such as artisanal tablets, which can contain up to seven classes of excipients (diluents/fillers, binders, disintegrants, lubricants, glidants, colorants, and preservatives) and sometimes complex mixtures of adulterants. Additionally, diclofenac, a nonsteroidal anti-inflammatory drug, and carisoprodol, a medication used for musculoskeletal pain, were found in the seized samples mixed with SCs (Table S3), along with several unidentified diluents. Despite some of these substances being electrochemically active, they did not interfere with the specific signals of SCs, demonstrating the robustness of the electrochemical method.

## Conclusions

The DPV technique with BDDE demonstrates, for the first time, the presence of a second cathodic process (R_2_) in the electrochemical behavior of SCs. This, along with the R_1_ process, contributes to the selective screening of these illicit substances in the seized samples. The proposed method offers high selectivity and sensitivity for detecting SCs in forensic samples using DPV with anodic and cathodic scanning on BDDE. Additionally, the DPV-BDDE method shows advantages over the Zimmermann colorimetric test for the preliminary identification of SCs in forensic analysis.

The method successfully classified three SCs—bupropion, benzylone, and dipentylone—that were not among the 15 SCs initially selected for the study, demonstrating robustness and specificity in detecting SCs in complex matrices. The generic identification of SC groups using the DPV-BDDE method can provide new insights for police investigations by enabling the correlation of trafficking flows with the locations where these drugs were seized.

Overall, the proposed method offers a rapid, simple, portable, robust, and selective test for SC groups in forensic analyses, providing a comprehensive solution for generic screening across the entire SC class.

## Supporting Information Available

Figures for pH, scan rate, CVs, and DPVs of SCs, PCA, and HCA analyses, mechanisms, and tables of data (PDF)
