# Supplementary material for: Synthetic Cathinones' Comprehensive Screening and Classification by Voltammetric and Chemometric Analyses: A Powerful Method for On-Site Forensic Applications
Source: Anal Chem. 2024 Oct 25;96(44):17746–53. doi: 10.1021/acs.analchem.4c04059 (PMC11541890; doi:10.1021/acs.analchem.4c04059)
Supplement: Supplementary file 1 — ac4c04059_si_001.pdf [file ac4c04059_si_001.pdf]

Synthetic Cathinones Comprehensive Screening and Classification by Voltammetric and Chemometric Analyses: A Powerful Method for On-Site Forensic Applications

Camila D. Lima[a], Luciano C. Arantes[b]*, Lara L. Machado[c], Thiago, R. L. C. Paixão[d], Wallans T. P. dos Santos[c]*

[a]Departamento de Química, [c]Departamento de Farmácia, Universidade Federal dos Vales do Jequitinhonha e Mucuri, Campus JK, 39100-000, Diamantina, Minas Gerais, Brazil.

[b]Laboratório de Química e Física Forense, Instituto de Criminalística, Polícia Civil do Distrito Federal, 70610-907, Brasília, Distrito Federal, Brazil.

[d]Departamento de Química Fundamental, Instituto de Química, Universidade de São Paulo, São Paulo, SP, 05508-000, Brazil.

*Corresponding Authors:

Emails: wallanst@ufvjm.edu.br and lca1969@gmail.com

Table of Contents

Figure S1. Generic representation of the colorimetric reaction between the Zimmermann's reagent and synthetic cathinones (SCs) derivatives. S5

Table S1. General information on the synthetic cathinones (SCs) studied and their classification into groups according to the proposed method....................................... S6

RESULTS AND DISCUSSION.. S9

Figure S2. (A) CVs on BDDE of 500 µmol L^{-1} *N*-alkylated SCs in 0.1 mol L^{-1} BR buffer at pH 8.0 (green line). All scans started at 0 V and proceeded in the cathodic direction at a scan rate of 50 mV s^{-1}. DPVs on BDDE of 100 µmol L^{-1} *N*-alkylated SCs in 0.1 mol L^{-1} BR at pH values ranging from 2.0 to 12.0, using anodic **(B)** and cathodic **(C)** scans. Experimental conditions: 80 mV amplitude, 10 mV step potential, 50 ms modulation time, and 0.1 s time interval. S9

Figure S3. (A) CVs on BDDE of 500 µmol L^{-1} 3,4-methylenedioxy-*N*-alkyl SCs in 0.1 mol L^{-1} BR buffer at pH 8.0 (green line). All scans started at 0 V and proceeded in the cathodic direction at a scan rate of 50 mV s^{-1}. DPVs on BDDE of 100 µmol L^{-1} 3,4-methylenedioxy-*N*-alkyl SCs in 0.1 mol L^{-1} BR at pH values ranging from 2.0 to 12.0, using anodic **(B)** and cathodic **(C)** scans. Experimental conditions: 80 mV amplitude, 10 mV step potential, 50 ms modulation time, and 0.1 s time interval. S10

Figure S4. (A) CVs on BDDE of 500 µmol L^{-1} 3,4-methylenedioxy-*N*-alkyl SCs in 0.1 mol L^{-1} BR buffer at pH 8.0 (green line). All scans started at 0 V and proceeded in the cathodic direction at a scan rate of 50 mV s^{-1}. DPVs on BDDE of 100 µmol L^{-1} 3,4-methylenedioxy-*N*-alkyl SCs in 0.1 mol L^{-1} BR at pH values ranging from 2.0 to 12.0, using anodic **(B)** and cathodic **(C)** scans. Experimental conditions: 80 mV amplitude, 10 mV step potential, 50 ms modulation time, and 0.1 s time interval. S11

Figure S5. (A) CVs on BDDE of 500 µmol L^{-1} *N* -pyrrolidine SCs in 0.1 mol L^{-1} BR buffer at pH 8.0 (green line). All scans started at 0 V and proceeded in the cathodic direction at a scan rate of 50 mV s^{-1}. DPVs on BDDE of 100 µmol L^{-1} *N* -pyrrolidine SCs in 0.1 mol L^{-1} BR at pH values ranging from 2.0 to 12.0, using anodic **(B)** and cathodic **(C)** scans. Experimental conditions: 80 mV amplitude, 10 mV step potential, 50 ms modulation time, and 0.1 s time interval... S12

Figure S6. (A) CVs on BDDE of 500 µmol L^{-1} 3,4-methylenedioxy-*N*-pyrrolidine SCs in 0.1 mol L^{-1} BR buffer at pH 8.0 (green line). All scans started at 0 V and proceeded in

the cathodic direction at a scan rate of 50 mV s^{-1}. DPVs on BDDE of 100 µmol L^{-1} 3,4-methylenedioxy-*N*-pyrrolidine SCs in 0.1 mol L^{-1} BR at pH values ranging from 2.0 to 12.0, using anodic **(B)** and cathodic **(C)** scans. Experimental conditions: 80 mV amplitude, 10 mV step potential, 50 ms modulation time, and 0.1 s time interval. S13

Figure S7. (A) CV on BDDE of 500 µmol L^{-1} ring thiophene *N*-pyrrolidine SC in 0.1 mol L^{-1} BR buffer at pH 8.0 (green line). All scans started at 0 V and proceeded in the cathodic direction at a scan rate of 50 mV s^{-1}. DPVs on BDDE of 100 µmol L^{-1} ring thiophene *N*-pyrrolidine SC in 0.1 mol L^{-1} BR at pH values ranging from 2.0 to 12.0, using anodic **(B)** and cathodic **(C)** scans. Experimental conditions: 80 mV amplitude, 10 mV step potential, 50 ms modulation time, and 0.1 s time interval. S14

Figure S8. (A) PCA score graph and (B) 3D scatter plot of the 15 SC standards, analyzed by DPV in BR buffer (0.1 mol L^{-1}, pH 8.0). Experimental conditions: 80 mV amplitude, 10 mV step potential, 50 ms modulation time, and 0.1 s time interval. S15

Figure S9. CVs of 500 µmol L^{-1} SCs in 0.1 mol L^{-1} BR buffer, pH 8.0, on BDDE. All potential sweeps started at 0.0 V in the cathodic direction, as indicated by the arrow, with sweep rates (v) ranging from 10 mV s^{-1} to 800 mV s^{-1}. Insets show linear regressions of *log ip vs. log v*. S16

Figure S10. CVs of 500 µmol L^{-1} SCs in 0.1 mol L^{-1} BR buffer, pH 8.0, on BDDE. All potential sweeps started at 0.0 V in the cathodic direction, as indicated by the arrow, with sweep rates (v) ranging from 10 mV s^{-1} to 800 mV s^{-1}. Insets show linear regressions of *log ip vs. log v*. S17

Pretreatment studies on the BDDE surface for SCs detection S18

Figure S11. CVs recorded in 0.1 mol L-1 BR buffer at pH 8.0 with 500 µmol L^{-1} MPHP, using BDDE without pretreatment (magenta-line), with anodic pretreatment (black-line), and with cathodic pretreatment (red-line). All potential scans were started at 0.0 V with a scan rate of 50 mV $s^{-1.}$ S18

Figure S12. Dendrogram generated using the HCA algorithm and Euclidean distances for fifteen SCs, based on DPV data obtained with BDDE. S19

Figure S13. DPVs on BDDE of 100 µmol L^{-1} SCs from group G1 (A), and group G2 (B-C) in 0.1 mol L−1 BR buffer at pH 8.0, using anodic (red arrow) and cathodic (blue arrow) scans. Experimental conditions: 80 mV amplitude, 10 mV step potential, 50 ms modulation time, and 0.1 s time interval. S20

Figure S14. DPVs on BDDE of 100 µmol L^{-1} SCs from group G3 (A), group G4 (B), group G5 (C) and group G6 (D) in 0.1 mol L−1 BR buffer at pH 8.0, using anodic (red

arrow) and cathodic (blue arrow) scans. Experimental conditions: 80 mV amplitude, 10 mV step potential, 50 ms modulation time, and 0.1 s time interval. S21
Mechanistic proposals for the anodic and cathodic processes of the SC groups .. S22
Figure S15. Proposed oxidation mechanism for SCs derivatives in the G1 group...... S22
Figure S16. Proposed oxidation mechanism for SCs derivatives in the G2 group...... S23
Figure S17. Proposed oxidation mechanism for SCs derivatives in the G5 group...... S24
Figure S18. Proposed reduction mechanism for all investigated SCs. S25
Table S2. Intra-day and inter-day repeatability (N = 5) for the R_1 process of 100 µmol L^{-1} SC standards. S26
Pulsed voltammetric technique optimization S27
Figure S19. (A) DPVs on BDDE of 15 – 100 µmol L^{-1} MPHP and (B) its respective calibration curve: I (µA) = - 0.42 (± 0.06) - 0.048 (± 0.001) [MPHP] (µA / µmol L^{-1}). (C) SWVs on BDDE of 25.5 – 76 µmol L^{-1} MPHP obtained by cathodic scans and (D) its respective calibration curve: I (µA) = - 0.32 (± 0.06) - 0.052 (± 0.002) [MPHP] (µA / µmol L^{-1}). Experimental conditions: 0.1 mol L^{-1} BR buffer at pH 8.0 was used as supporting electrolyte for both techniques, with current peaks obtained from R_1 using cathodic scans. Optimized parameters for DPV: 80 mV amplitude, 10 mV step potential, 50 ms modulation time, and 0.1 s time interval; for SWV: 50 mV amplitude, 20 HZ Frequency and 5 mV step potential. S28
Figure S20. DPVs on BDDE using anodic (A) and cathodic (B) scans in the presence of SCs representatives from groups G1 to G6 and interferents such as caffeine, paracetamol, and anesthetic medications. S29
Figure S21. DPVs using anodic and cathodic scans with baseline correction, obtained from screening SCs in real samples diluted 800x in 0.1 mol L^{-1} BR buffer at pH 8.0. Experimental conditions: 80 mV amplitude, 10 mV step potential, 50 ms modulation time, and 0.1 s time interval; anodic sweep potential window from - 1.9 to + 2.0 V and cathodic sweep from + 2.0 to - 1.9 V. S30
Figure S22. DPVs using anodic and cathodic scans without baseline correction, obtained from real sample 4 diluted 800x in 0.1 mol L^{-1} BR buffer at pH 8.0. The experimental conditions were the same as in Figure S21. .... S31
Table S3. Comparison of results from the DPV-BDDE screening method for SCs with LC-MS results for seized samples. S32
REFERENCES S33

Supporting Information

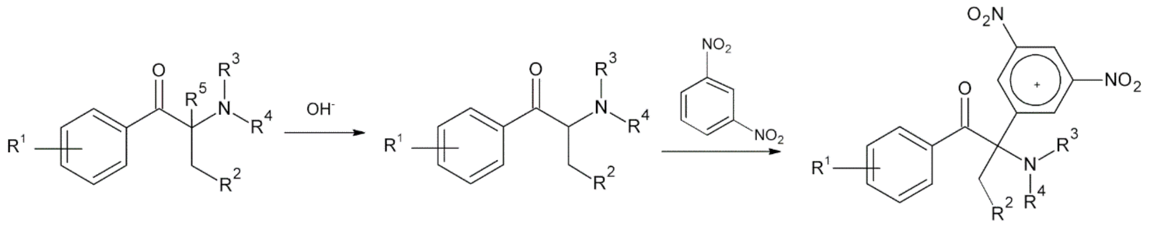

Figure S1. Generic representation of the colorimetric reaction between the Zimmermann's reagent and synthetic cathinones (SCs) derivatives.

Table S1. General information on the synthetic cathinones (SCs) studied and their classification into groups according to the proposed method.

(continued)

Synthetic cathinone derivatives												
**Common name**	**IUPAC Name**	**Chemical formula**	**CAS Number**	**R1**	**R2**	**R3**	**R4**	**R5**	**MW**	**ANVISA Schedulling List**	**Chemical structure**	**SC Group**
**Ethcathinone**; N-Ethylcathinone; Ethylaminopropiophenone; Ethylpropion; ETH-CAT; 2-(Ethylamino)propiophenone	2-(Ethylamino)-1-phenyl-1-propanone	$C_{11}H_{15}NO$	51553-17-4	H	H	H	C_2H_5	H	177,24	Lista F2 "c"	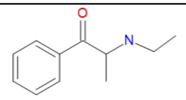	1
4-MMC; **Mephedrone**; 4-MeMC; 4-Methylephedrone; 4-Methylmethcathinone	2-(Methylamino)-1-(4-methylphenyl)-1-propanone	$C_{11}H_{15}NO$	1189726-22-4	CH_3	H	H	CH_3	H	177,24	Lista F2 "a"	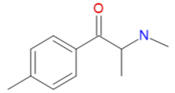	1
**4-MPD**; 4-methyl Pentedrone; 4-Methylpentedrone; 4-methyl-α-methylamino-Valerophenone	2-(Methylamino)-1-(4-methylphenyl)-1-pentanone	$C_{13}H_{19}NO$	1373918-61-6	CH_3	C_2H_5	H	CH_3	H	205,30	Lista F2 "c"	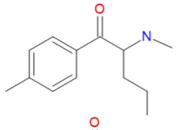	1
**Bupropion**	1-(3-Chlorophenyl)-2-[(2-methyl-2-propanyl)amino]-1-propanone	$C_{13}H_{18}ClNO$	31677-93-7	Cl	H	H	Tert-butyl	H	239,74	Lista C1	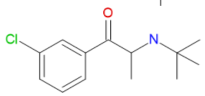	1
bk-MDMA; **Methylone**; 3,4-Methylenedioxy-N-methylcathinone; β-keto Methylenedioxy Methamphetamine; 3,4-methylenedioxymethcathinone	1-(1,3-Benzodioxol-5-yl)-2-(methylamino)-1-propanone	$C_{11}H_{13}NO_3$	186028-80-8	3,4-Methylenedioxy	H	H	CH_3	H	207,23	Lista F2 "a"	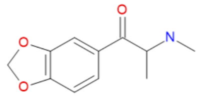	2
bk-MDEA; **Ethylone**; MDEC; bk-Methylenedioxyethylamphetamine; 3,4-Methylenedioxy-N-ethylcathinone	1-(1,3-Benzodioxol-5-yl)-2-(ethylamino)-1-propanone	$C_{12}H_{15}NO_3$	1454266-19-3	3,4-Methylenedioxy	H	H	C_2H_5	H	221,25	Lista F2 "a"	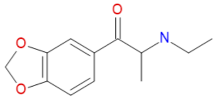	2
bk-EBDB; **Eutylone**; β-keto-Ethylbenzodioxolylbutanamine; Ethyl Butylone; N-ethyl Butylone; 3,4-Methylenedioxy-α-Ethylaminobutiophenone	1-(1,3-Benzodioxol-5-yl)-2-(ethylamino)-1-butanone	$C_{13}H_{17}NO_3$	17764-18-0	3,4-Methylenedioxy	CH_3	H	C_2H_5	H	235,28	Lista F2 "a"	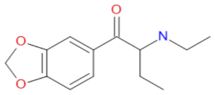	2

Synthetic cathinone derivatives

Common name	IUPAC Name	Chemical formula	CAS Number	R1	R2	R3	R4	R5	MW	ANVISA Schedulling List	Chemical structure	SC Group
N-Ethylpentylone; **Ephylone**; N-ethyl Pentylone	1-(1,3-Benzodioxol-5-yl)-2-(ethylamino)-1-pentanone	$C_{14}H_{19}NO_3$	17763-02-9	3,4-Methylenedioxy	C_2H_5	H	C_2H_5	H	249,31	Lista F2 "a"	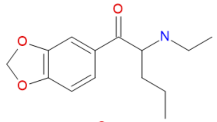	2
**MDPT; 3',4'-Methylenedioxy-N-tert-butylcathinone; tBuONE; D-Tertylone**	1-(1,3-Benzodioxol-5-yl)-2-[(2-methyl-2-propanyl)amino]-1-propanone	C14H19NO3	2469270-98-0	3,4-Methylenedioxy	H	H	Tert-butyl	H	249,31	Lista F2 "c"	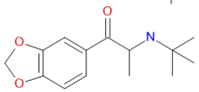	2
BMDP; **Benzylone**; 3,4-Methylenedioxy-N-benzylcathinone	1-(1,3-benzodioxol-5-yl)-2-[(phenylmethyl)amino]-1-propanone	$C_{17}H_{17}NO_3$	1823274-68-5	3,4-Methylenedioxy	H	H	Phenyl	H	283,32	Lista F2 "c"	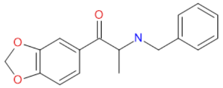	2
bk-DMBDB; **Dibutylone**; bk-MMBDB; Methylbutylone	1-(1,3-Benzodioxol-5-yl)-2-(dimethylamino)-1-butanone	$C_{13}H_{17}NO_3$	17763-12-1	3,4-Methylenedioxy	CH_3	CH_3	CH_3	H	235,28	Lista F2 "a"	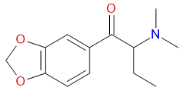	3
bk-DMBDP; **Dipentylone**; N-N,Dimethylpentylone; Dimethylpentylone	1-(1,3-Benzodioxol-5-yl)-2-(dimethylamino)-1-pentanone	$C_{14}H_{19}NO_3$	17763-13-2	3,4-Methylenedioxy	C_2H_5	CH_3	CH_3	H	249,31	Lista F2 "c"	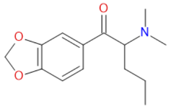	3
**MDPV**; 3,4-Methylenedioxy Pyrovalerone; 3,4-MDPV; Methylenedioxypyrovalerone; 3,4-Methylenedioxypyrovalerone	1-(1,3-Benzodioxol-5-yl)-2-(1-pyrrolidinyl)-1-pentanone	$C_{16}H_{21}NO_3$	24622-62-6	3,4-Methylenedioxy	C_2H_5	Pyrrolidinyl		H	275,34	Lista F1	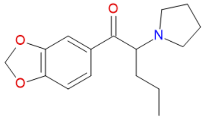	4
**MDPHP**; 3,4-Methylenedioxy-α-Pyrrolidinohexaphenone; 3,4-MD-α-PHP; 3,4-MDPHP; 3,4-Methylenedioxy-α-Pyrrolidinohexiophenone; Methylenedioxypyrrolidinohexiophenone	1-(1,3-Benzodioxol-5-yl)-2-(1-pyrrolidinyl)-1-hexanone	$C_{17}H_{23}NO_3$	24622-61-5	3,4-Methylenedioxy	C_3H_7	Pyrrolidinyl		H	289,37	Lista F2 "c"	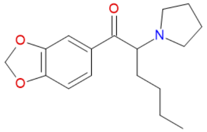	4

Synthetic cathinone derivatives												
**Common name**	**IUPAC Name**	**Chemical formula**	**CAS Number**	**R1**	**R2**	**R3**	**R4**	**R5**	**MW**	**ANVISA Schedulling List**	**Chemical structure**	**SC Group**
**α-PVP**; α-Pyrrolidinopentiophenone; α-Pyrrolidinovalerophenone; α-Pyrrolidinopentiphenone	1-Phenyl-2-(1-pyrrolidinyl)-1-pentanone	$C_{15}H_{21}NO$	5485-65-4	H	C_2H_5	Pyrrolidinyl		H	231,33	Lista F2 "a"	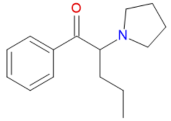	5
**MPHP**; 4'-methyl-α-Pyrrolidinohexanophenone; 4'-methyl-α-PHP; PV4; PV-4; 4'-methyl-α-Pyrrolidinohexiophenone	1-(4-Methylphenyl)-2-(1-pyrrolidinyl)-1-hexanone	$C_{17}H_{25}NO$	1391052-36-0	CH_3	C_3H_7	Pyrrolidinyl		H	259.387	Lista F2 "c"	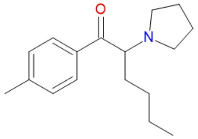	5
**TH-PVP**; 3',4'-Tetramethylene-α-Pyrrolidinovalerophenone; 3',4'-Tetramethylene-α-PVP	2-(1-Pyrrolidinyl)-1-(5,6,7,8-tetrahydro-2-naphthalenyl)-1-pentanone	$C_{19}H_{27}NO$	2748590-73-8	3,4-Tetramethylene	C_2H_5	Pyrrolidinyl		H	285,42	Lista F2 "a"	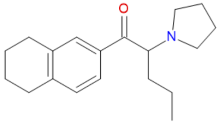	5
**α-PVT**; α-Pyrrolidinopentiophenone; α-Pyrrolidinovalerothiophenone	2-(1-Pyrrolidinyl)-1-(2-thienyl)-1-pentanone	$C_{13}H_{19}NO_S$	2748622-52-6	Thiopheno	C_2H_5	Pyrrolidinyl		H	237,36	Unschedulled	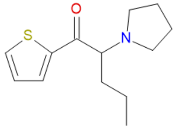	6

RESULTS AND DISCUSSION

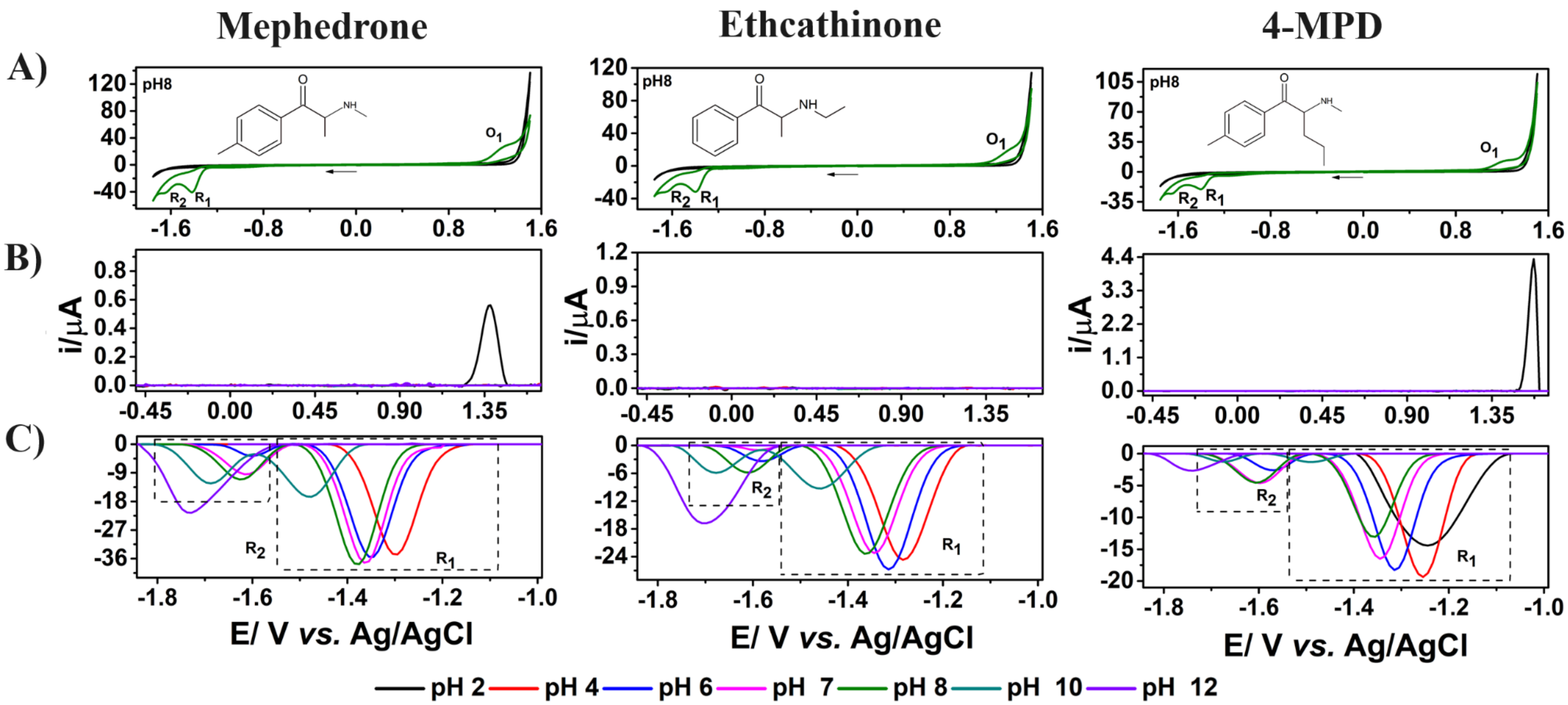

Figure S2. (A) CVs on BDDE of 500 μmol L^{-1} *N*-alkylated SCs in 0.1 mol L^{-1} BR buffer at pH 8.0 (green line). All scans started at 0 V and proceeded in the cathodic direction at a scan rate of 50 mV s^{-1}. DPVs on BDDE of 100 μmol L^{-1} *N*-alkylated SCs in 0.1 mol L^{-1} BR at pH values ranging from 2.0 to 12.0, using anodic **(B)** and cathodic **(C)** scans. Experimental conditions: 80 mV amplitude, 10 mV step potential, 50 ms modulation time, and 0.1 s time interval.

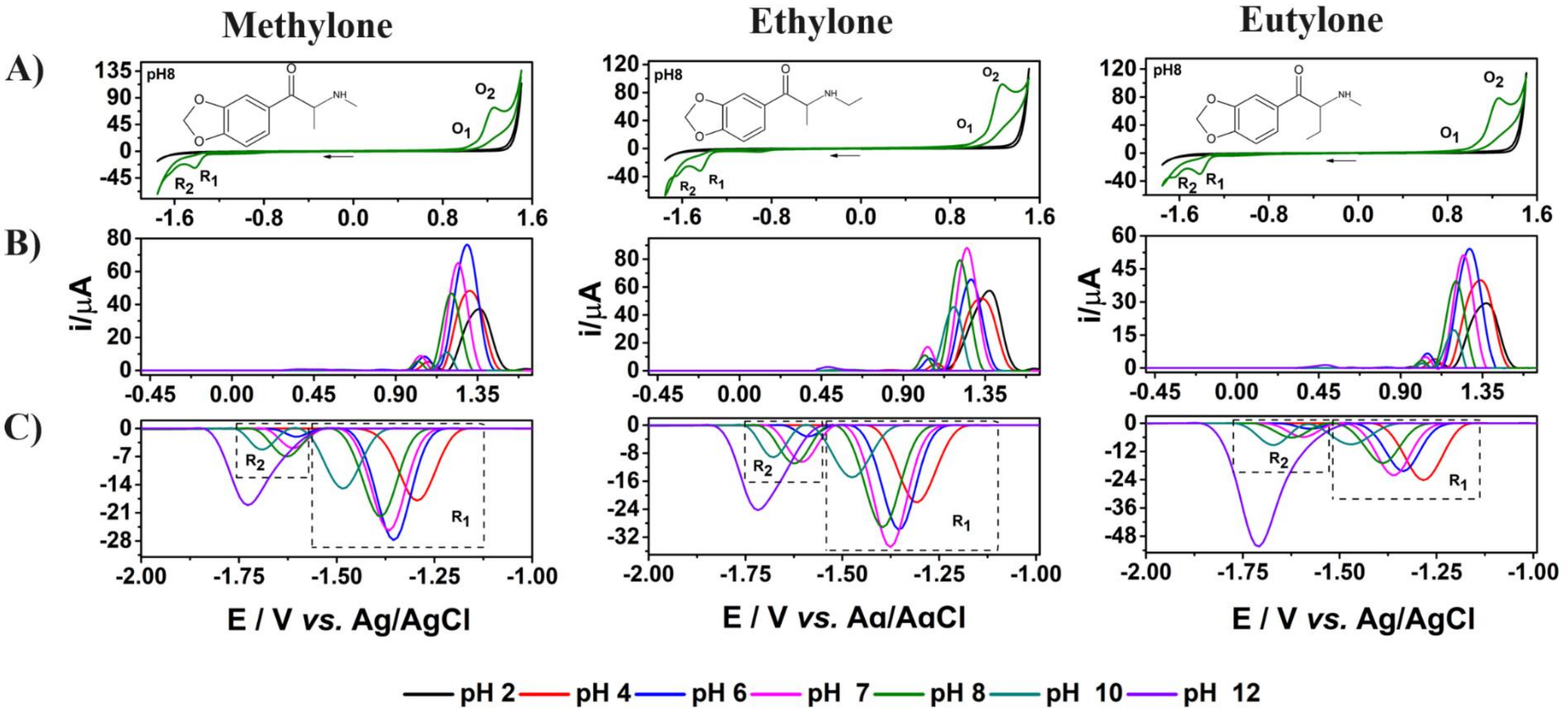

Figure S3. (A) CVs on BDDE of 500 μmol L^{-1} 3,4-methylenedioxy-*N*-alkyl SCs in 0.1 mol L^{-1} BR buffer at pH 8.0 (green line). All scans started at 0 V and proceeded in the cathodic direction at a scan rate of 50 mV s^{-1}. DPVs on BDDE of 100 μmol L^{-1} 3,4-methylenedioxy-*N*-alkyl SCs in 0.1 mol L^{-1} BR at pH values ranging from 2.0 to 12.0, using anodic **(B)** and cathodic **(C)** scans. Experimental conditions: 80 mV amplitude, 10 mV step potential, 50 ms modulation time, and 0.1 s time interval.

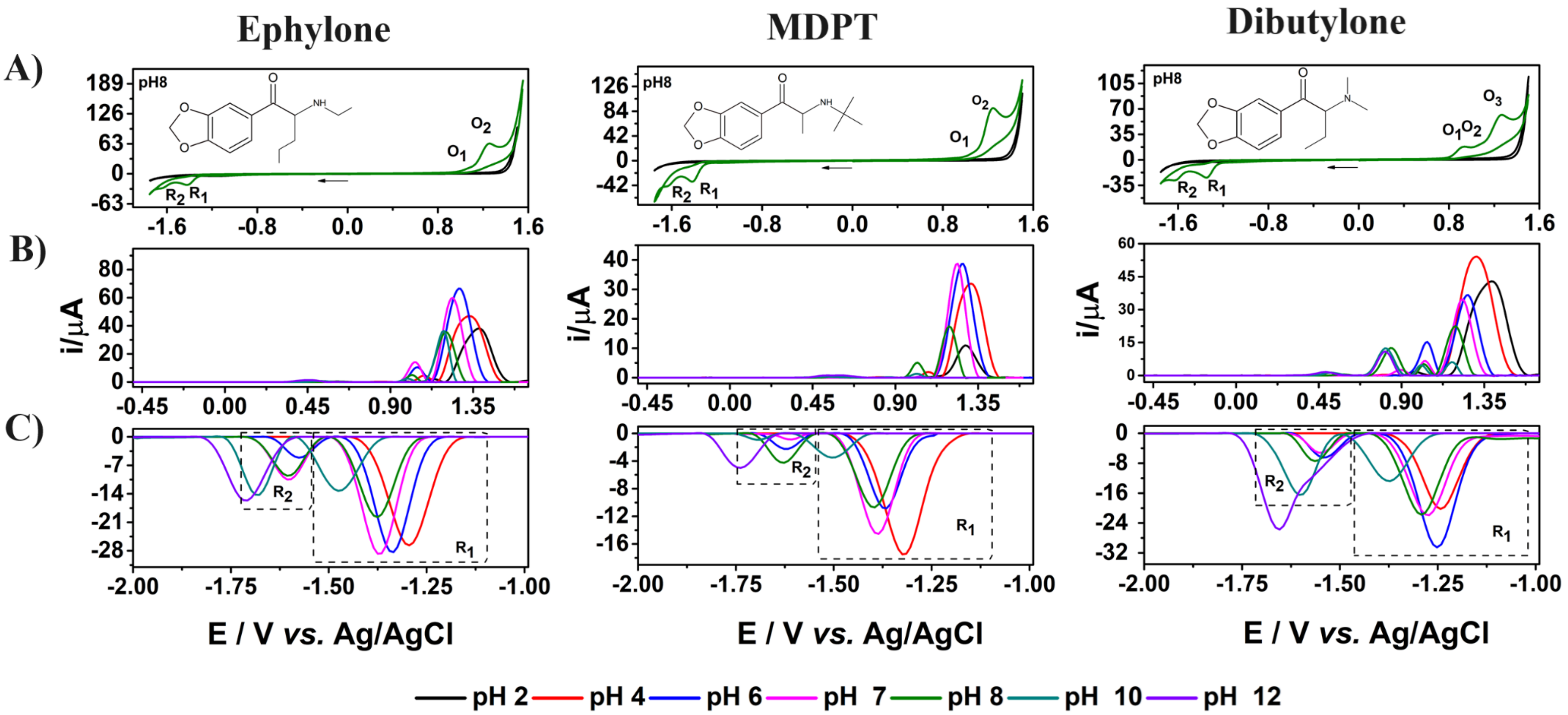

Figure S4. (A) CVs on BDDE of 500 μmol L^{-1} 3,4-methylenedioxy-*N*-alkyl SCs in 0.1 mol L^{-1} BR buffer at pH 8.0 (green line). All scans started at 0 V and proceeded in the cathodic direction at a scan rate of 50 mV s^{-1}. DPVs on BDDE of 100 μmol L^{-1} 3,4-methylenedioxy-*N*-alkyl SCs in 0.1 mol L^{-1} BR at pH values ranging from 2.0 to 12.0, using anodic **(B)** and cathodic **(C)** scans. Experimental conditions: 80 mV amplitude, 10 mV step potential, 50 ms modulation time, and 0.1 s time interval.

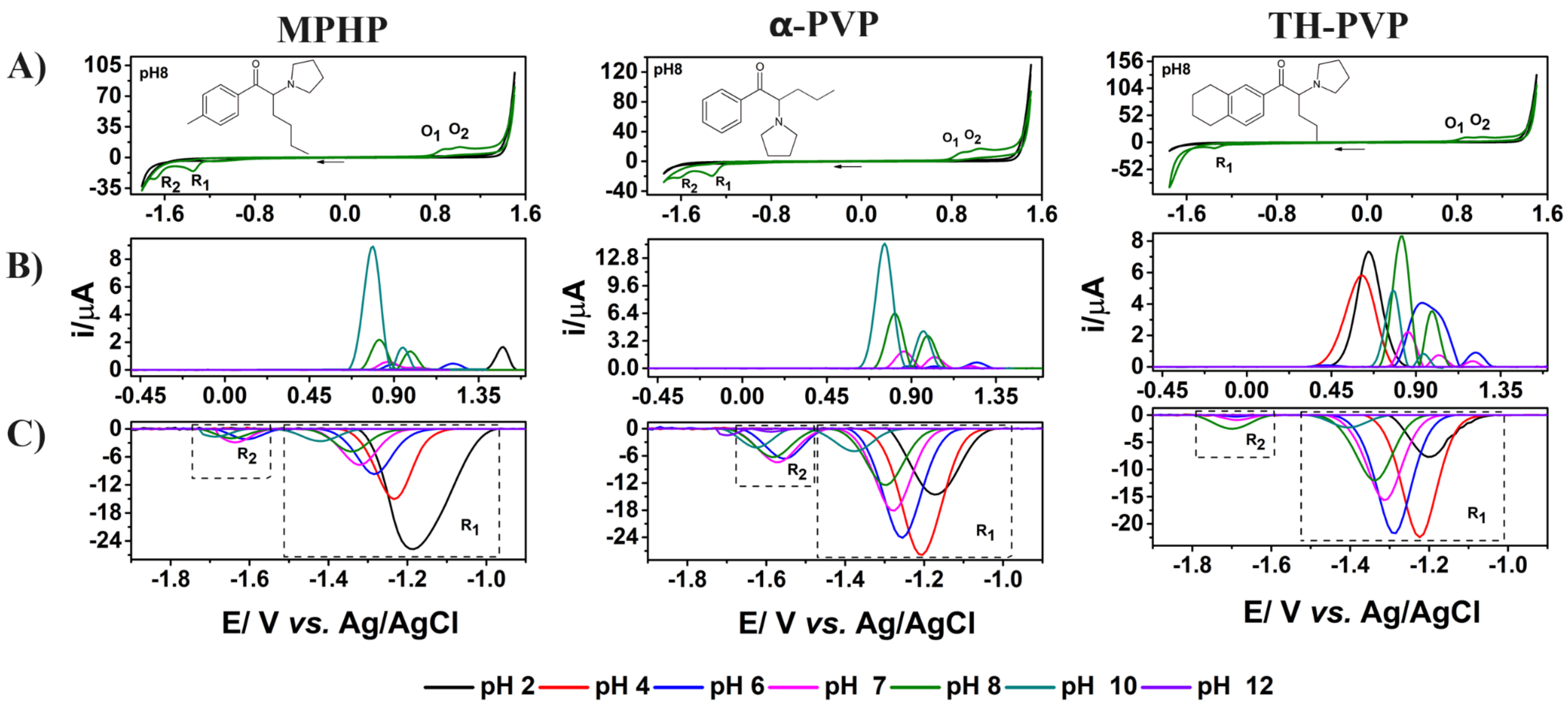

Figure S5. (A) CVs on BDDE of 500 μmol L^{-1} *N*-pyrrolidine SCs in 0.1 mol L^{-1} BR buffer at pH 8.0 (green line). All scans started at 0 V and proceeded in the cathodic direction at a scan rate of 50 mV s^{-1}. DPVs on BDDE of 100 μmol L^{-1} *N*-pyrrolidine SCs in 0.1 mol L^{-1} BR at pH values ranging from 2.0 to 12.0, using anodic **(B)** and cathodic **(C)** scans. Experimental conditions: 80 mV amplitude, 10 mV step potential, 50 ms modulation time, and 0.1 s time interval.

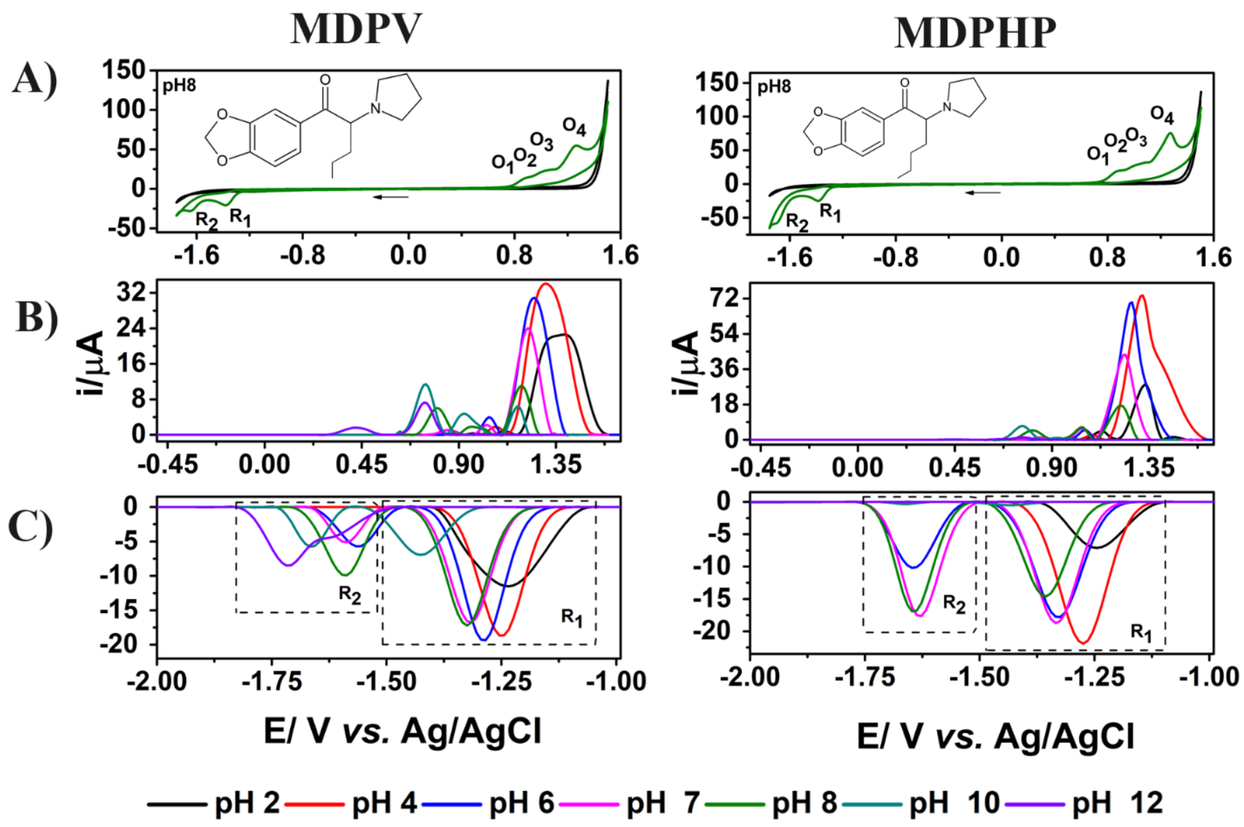

Figure S6. **(A)** CVs on BDDE of 500 μmol L^{-1} 3,4-methylenedioxy-*N*-pyrrolidine SCs in 0.1 mol L^{-1} BR buffer at pH 8.0 (green line). All scans started at 0 V and proceeded in the cathodic direction at a scan rate of 50 mV s^{-1}. DPVs on BDDE of 100 μmol L^{-1} 3,4-methylenedioxy-*N*-pyrrolidine SCs in 0.1 mol L^{-1} BR at pH values ranging from 2.0 to 12.0, using anodic **(B)** and cathodic **(C)** scans. Experimental conditions: 80 mV amplitude, 10 mV step potential, 50 ms modulation time, and 0.1 s time interval.

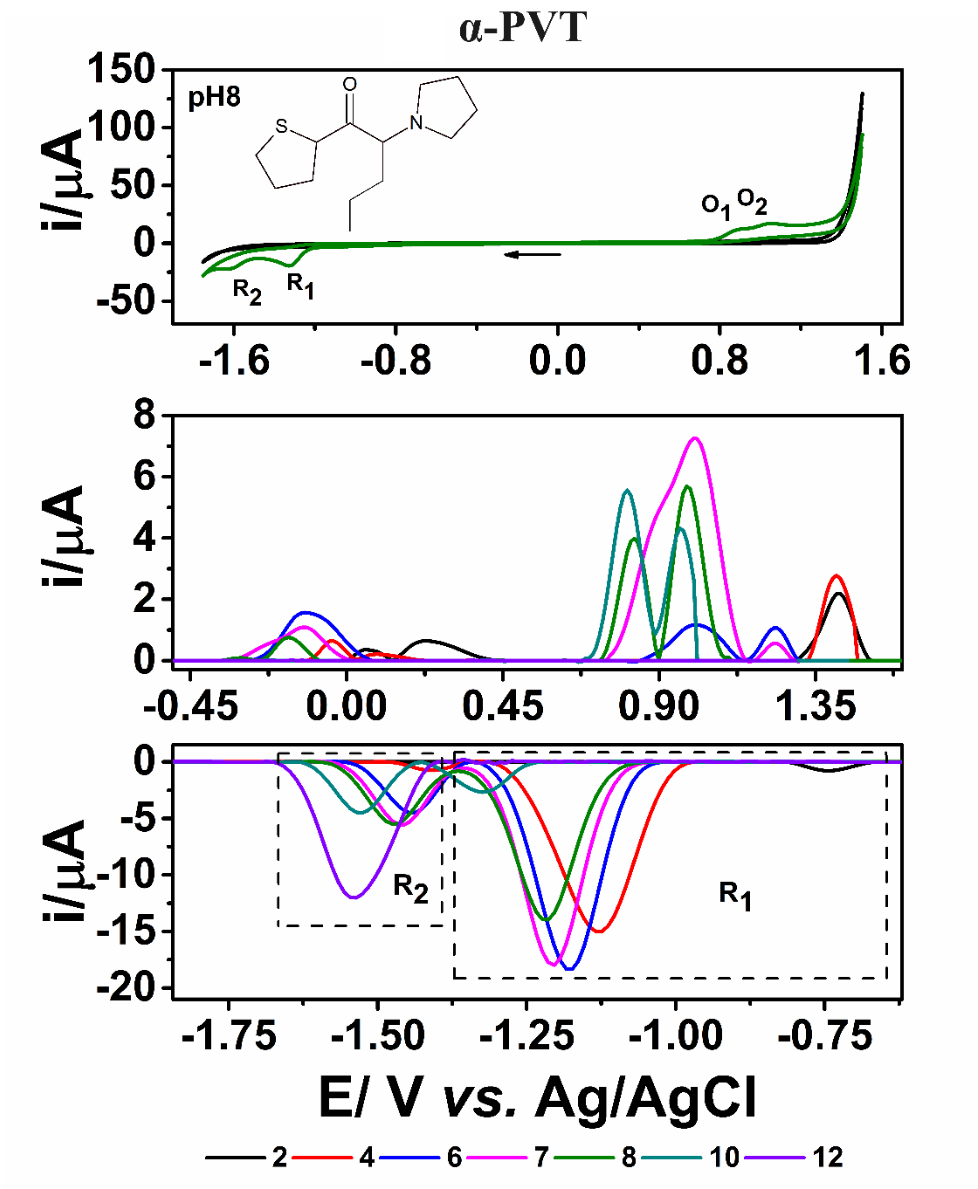

Figure S7. (A) CV on BDDE of 500 µmol L^{-1} ring thiophene *N*-pyrrolidine SC in 0.1 mol L^{-1} BR buffer at pH 8.0 (green line). All scans started at 0 V and proceeded in the cathodic direction at a scan rate of 50 mV s^{-1}. DPVs on BDDE of 100 µmol L^{-1} ring thiophene *N*-pyrrolidine SC in 0.1 mol L^{-1} BR at pH values ranging from 2.0 to 12.0, using anodic **(B)** and cathodic **(C)** scans. Experimental conditions: 80 mV amplitude, 10 mV step potential, 50 ms modulation time, and 0.1 s time interval.

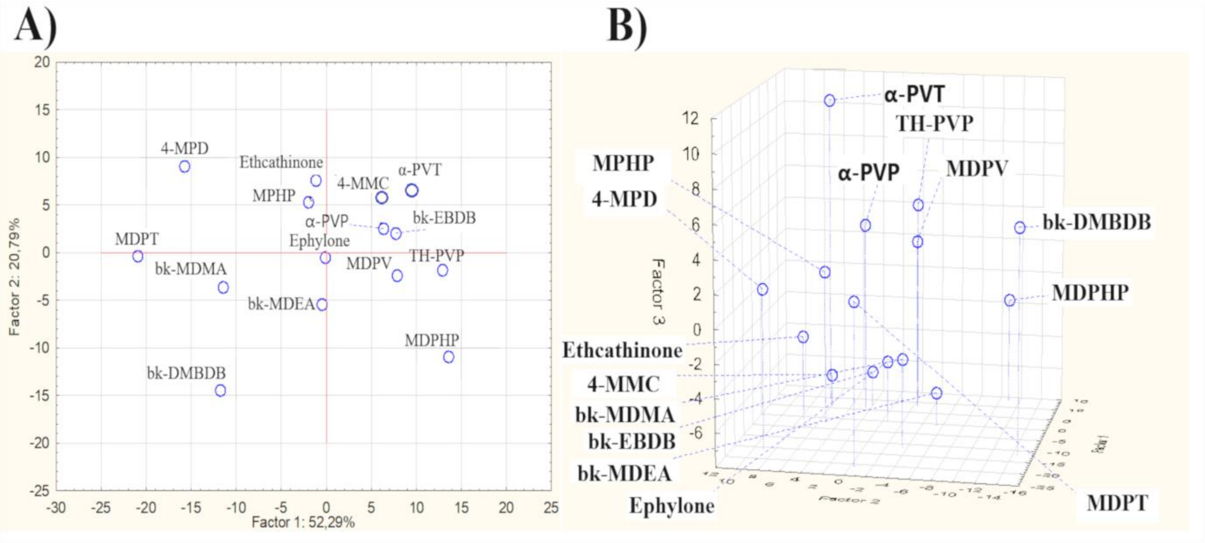

Figure S8. (A) PCA score graph and **(B)** 3D scatter plot of the 15 SC standards, analyzed by DPV in BR buffer (0.1 mol L^{-1}, pH 8.0). Experimental conditions: 80 mV amplitude, 10 mV step potential, 50 ms modulation time, and 0.1 s time interval.

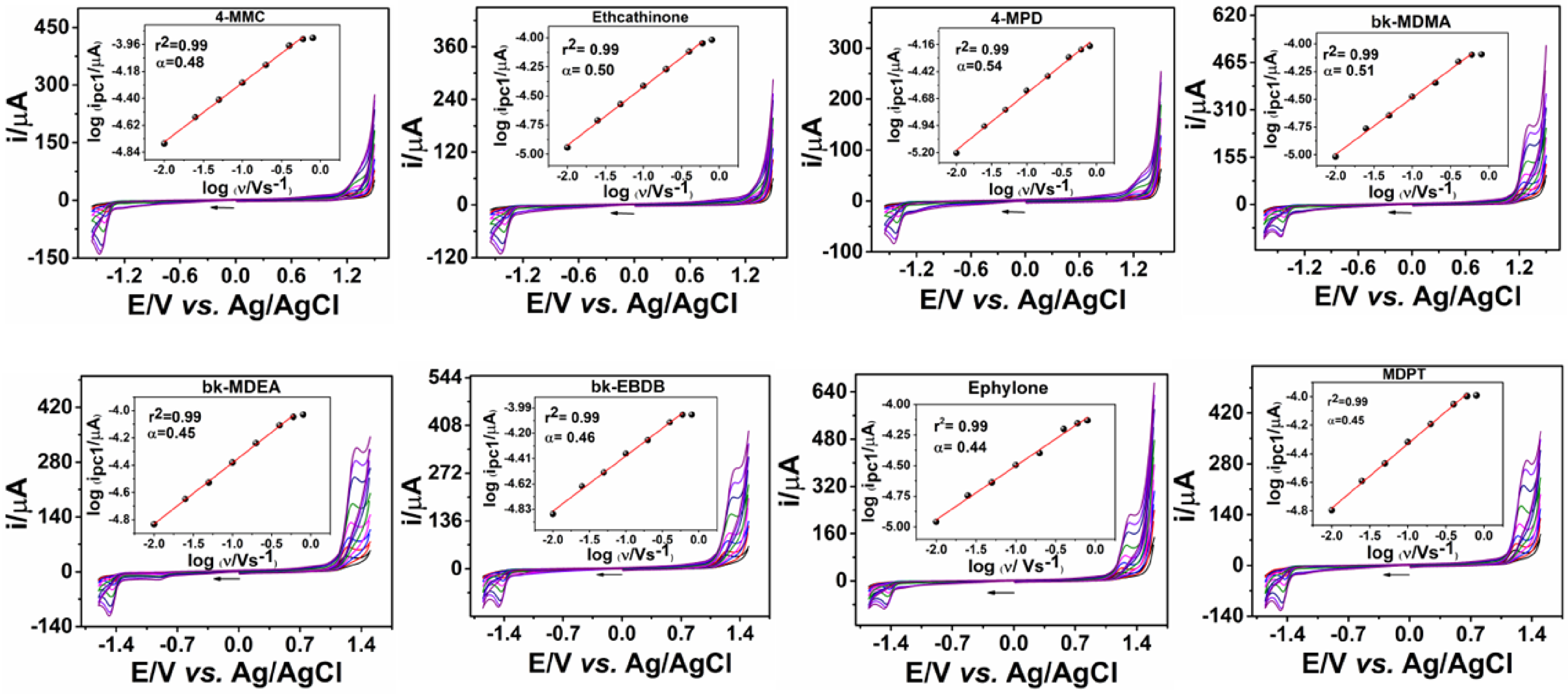

Figure S9. CVs of 500 µmol L^{-1} SCs in 0.1 mol L^{-1} BR buffer, pH 8.0, on BDDE. All potential sweeps started at 0.0 V in the cathodic direction, as indicated by the arrow, with sweep rates (v) ranging from 10 mV s^{-1} to 800 mV s^{-1}. Insets show linear regressions of *log ip vs. log v*.

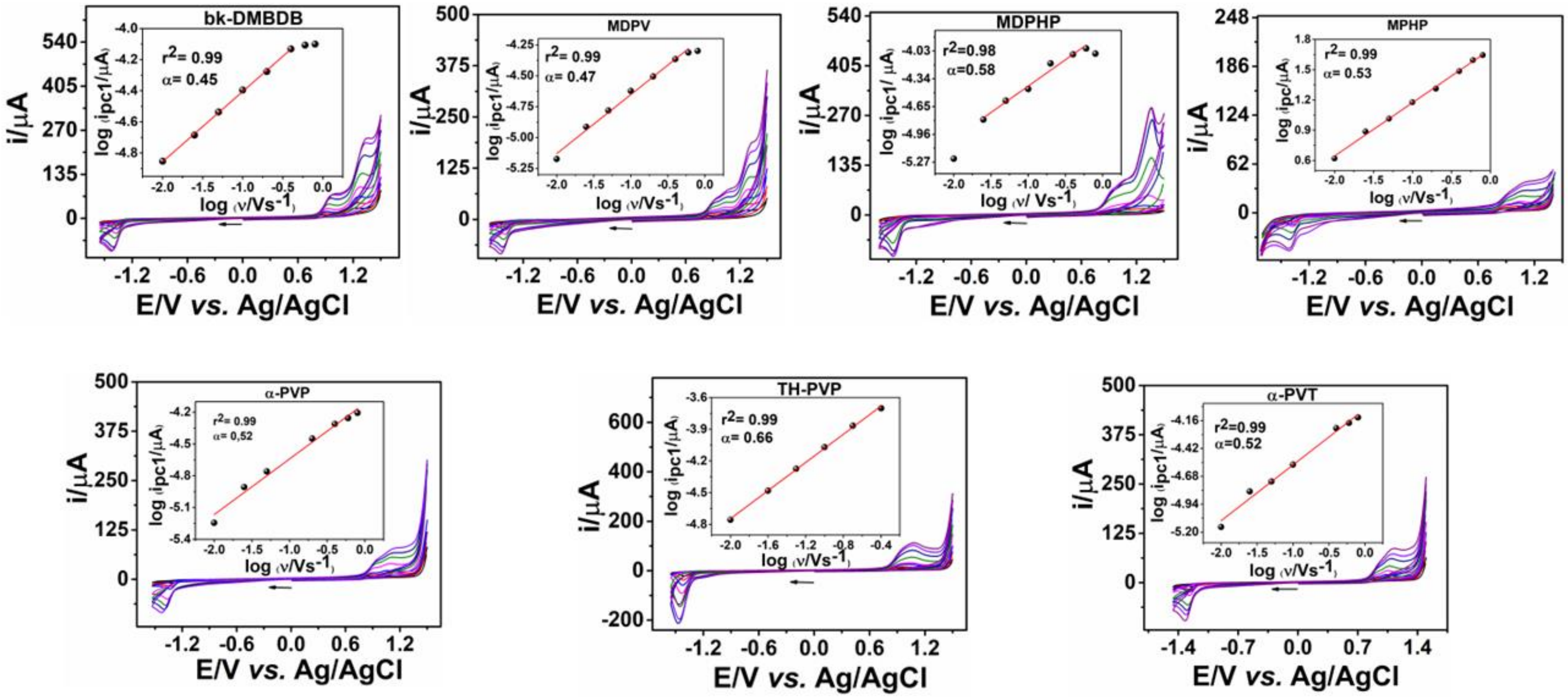

Figure S10. CVs of 500 µmol L^{-1} SCs in 0.1 mol L^{-1} BR buffer, pH 8.0, on BDDE. All potential sweeps started at 0.0 V in the cathodic direction, as indicated by the arrow, with sweep rates (v) ranging from 10 mV s^{-1} to 800 mV s^{-1}. Insets show linear regressions of *log ip vs. log v*.

Pretreatment studies on the BDDE surface for SCs detection

The surface treatment of BDDE can be either cathodic or anodic. In cathodic pretreatment, the surface becomes hydrogen-terminated and more hydrophobic due to water reduction. On the other hand, anodic pretreatment results in an oxygen-terminated, more hydrophilic surface due to water oxidation.[1,2] In this work, cathodic pretreatment was selected based on a previous study by our group,[3] where it provided enhanced electrochemical responses for phenylethylamines. Additionally, pretreatment studies (cathodic, anodic, and untreated) were performed on BDDE for detection of synthetic cathinones, using MPHP as the model molecule, as shown in Figure S11.

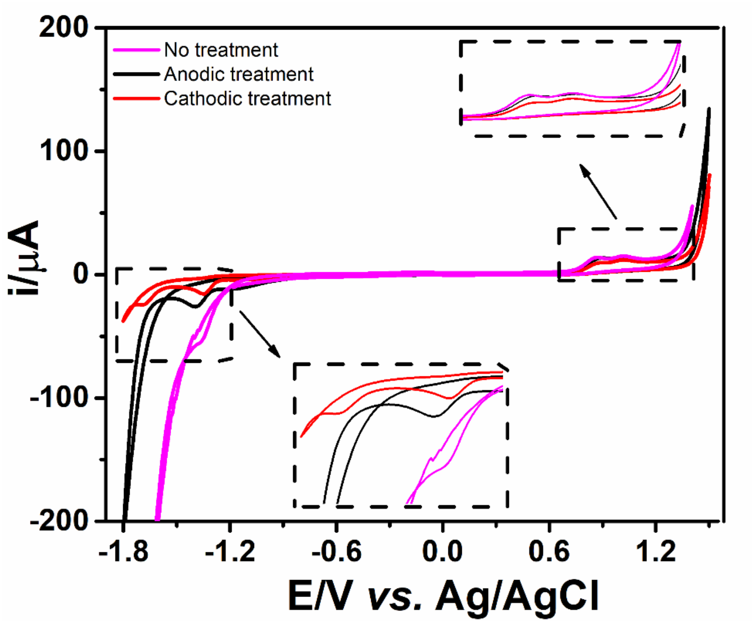

Figure S11. CVs recorded in 0.1 mol L-1 BR buffer at pH 8.0 with 500 µmol L^{-1} MPHP, using BDDE without pretreatment (magenta-line), with anodic pretreatment (black-line), and with cathodic pretreatment (red-line). All potential scans started at 0.0 V with a scan rate of 50 mV $s^{-1.}$

Figure S11 shows that the R_2 cathodic process of MPHP is only observed when cathodic pretreatment is applied to the BDDE. This is likely due to the surface becoming more hydrophobic after cathodic pretreatment, which shifts water discharge to more negative potentials, enabling the detection of the R_2 process for MPHP and other SCs. This observation is consistent with findings for another cathinone, as reported by Scheel *et al.*[4]

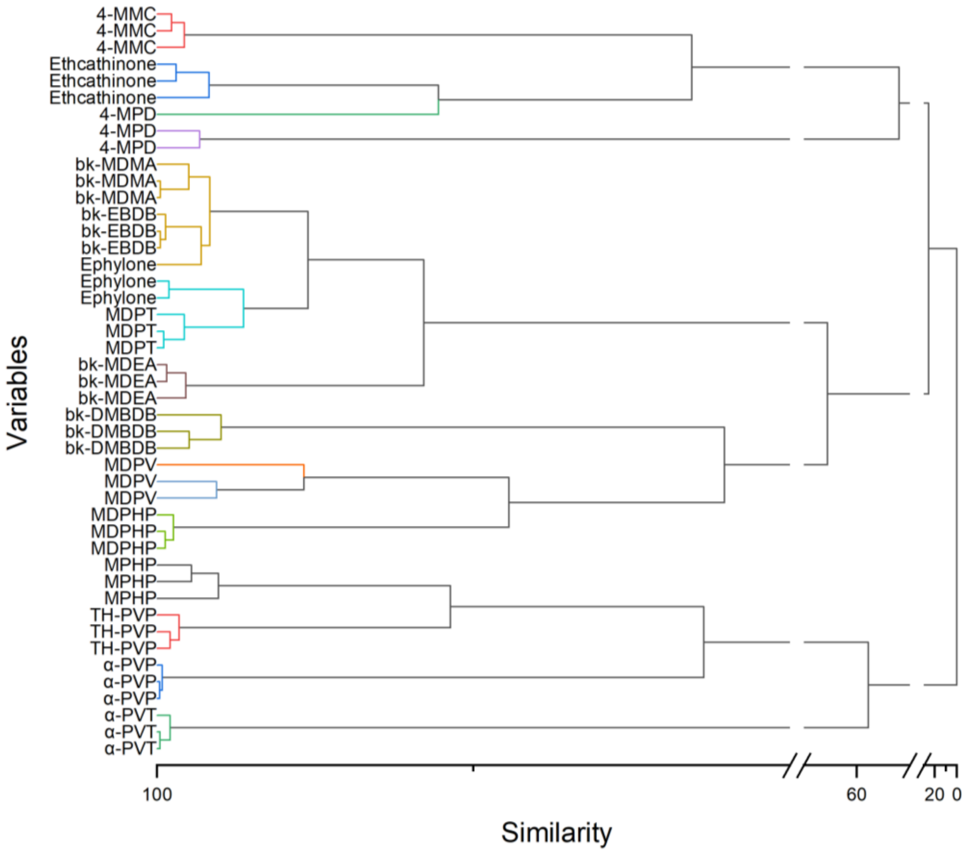

Figure S12. Dendrogram generated using the HCA algorithm and Euclidean distances for fifteen SCs, based on DPV data obtained with BDDE.

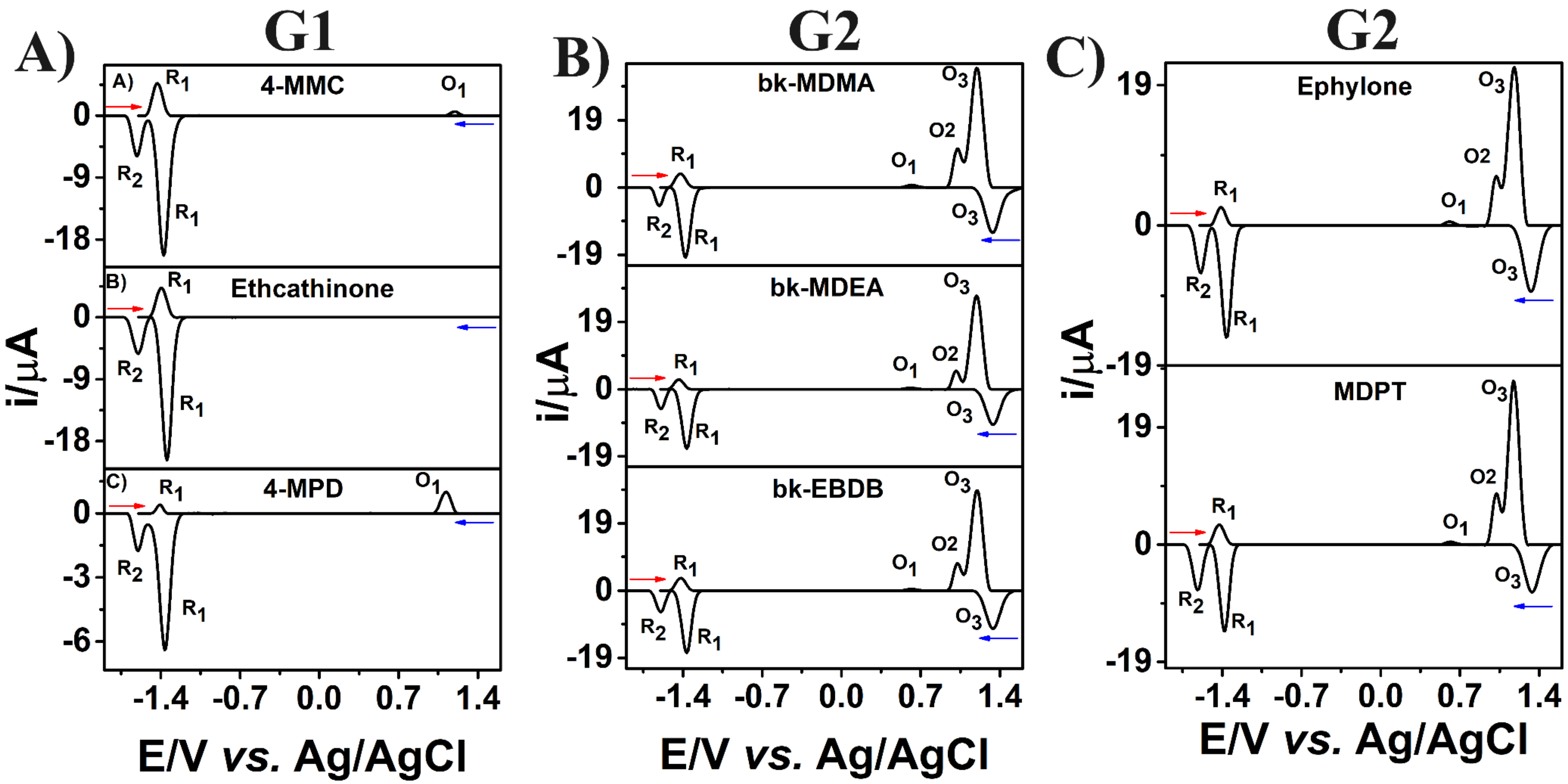

Figure S13. DPVs on BDDE of 100 μmol L^{-1} SCs from group G1 (A), and group G2 (B-C) in 0.1 mol L−1 BR buffer at pH 8.0, using anodic (red arrow) and cathodic (blue arrow) scans. Experimental conditions: 80 mV amplitude, 10 mV step potential, 50 ms modulation time, and 0.1 s time interval.

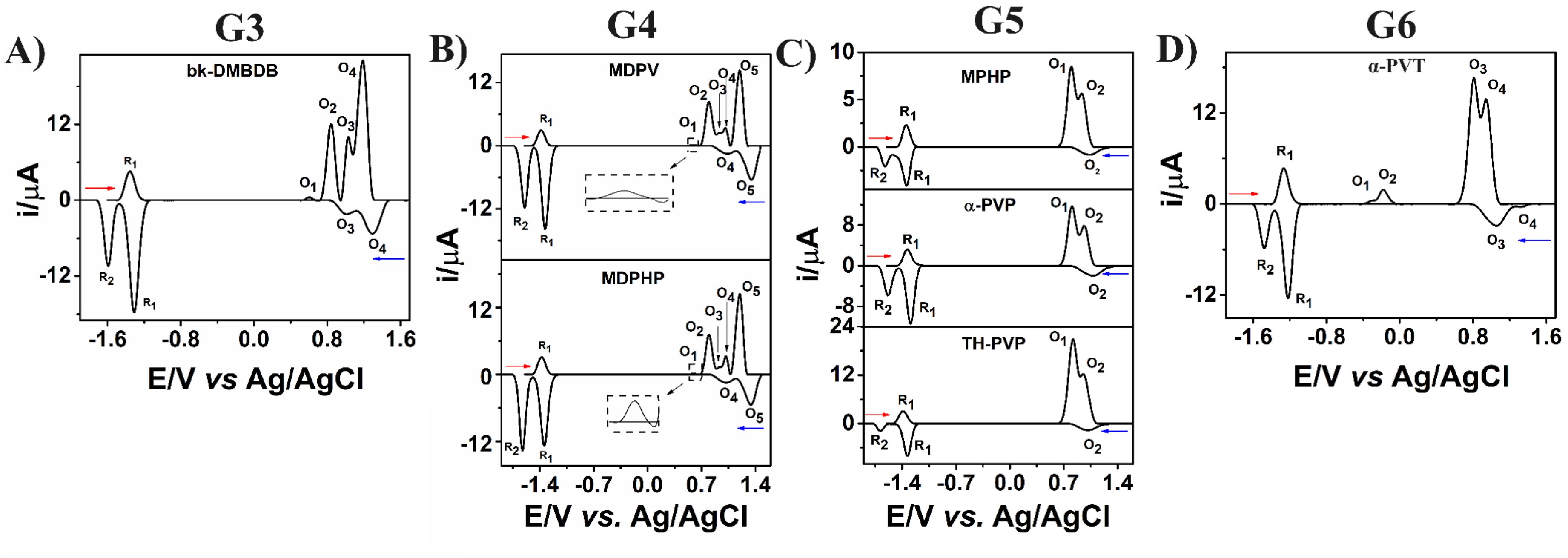

Figure S14. DPVs on BDDE of 100 μmol L^{-1} SCs from group G3 (A), group G4 (B), group G5 (C) and group G6 (D) in 0.1 mol L−1 BR buffer at pH 8.0, using anodic (red arrow) and cathodic (blue arrow) scans. Experimental conditions: 80 mV amplitude, 10 mV step potential, 50 ms modulation time, and 0.1 s time interval.

Mechanistic proposals for the anodic and cathodic processes of the SC groups

This section presents the mechanistic proposals for the electrochemical processes of the six SC groups on BDDE. It is worth mentioning that these proposals are based on previously reported work by Schram *et al.*[5], where the electrochemical behavior of synthetic cathinones such as mephedrone, ethcathinone, methylone, butylone, and 4-chloro-alpha-pyrrolidinovalerophenone (Cl-PVP) was prioritized. According to the classification proposed in our study, these SCs belong to groups G1, G2, and G5.

To confirm the redox products of these SCs, Schram *et al.*[5] performed analyses on electrolyzed solutions using high-performance liquid chromatography coupled with mass spectrometry (HPLC-MS). Based int their findings and our results, we propose that the O_I process of the G1 group SCs – analogous to methamphetamine – is related to the oxidation of the secondary amine, as previously suggested by Garrido *et al.*[6] Figure S15 shows the oxidation mechanism for the O_I process of G1 group SCs derivatives, as described for mephedrone on screen-printed carbon electrode (SPCE).[5]

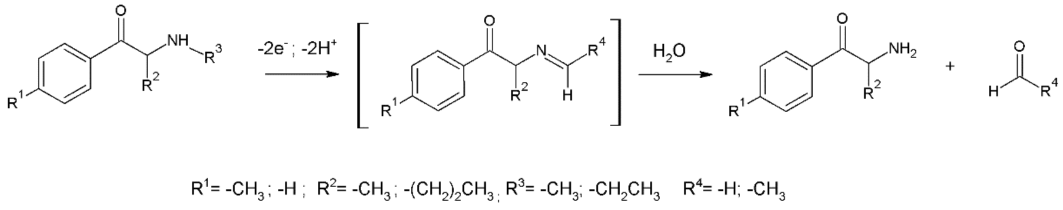

Figure S15. Proposed oxidation mechanism for SCs derivatives in the G1 group.

As shown in Figure S15, the oxidation of the secondary amine (1a) is a key step. Given that the G1 derivatives exhibit an electrochemical profile on BDDE similar to that of mephedrone on SPCE, this oxidation mechanism can be considered a generic proposal for SCs in the G1 group.

On the other hand, G2 group derivatives differ from those in the G1 group due to the presence of the 3,4-methylenedioxy ring, which introduces additional redox processes. Schram *et al.* also proposed an oxidation mechanism for methylone, and the resulting products were confirmed using HPLC-MS.[5] Figure S16 shows a generic mechanism for G2 group cathinones, adapted from Schram *et al.*[5] The generated products are correlated with the anodic processes observed by DPV in this work (Figure S13B-C).

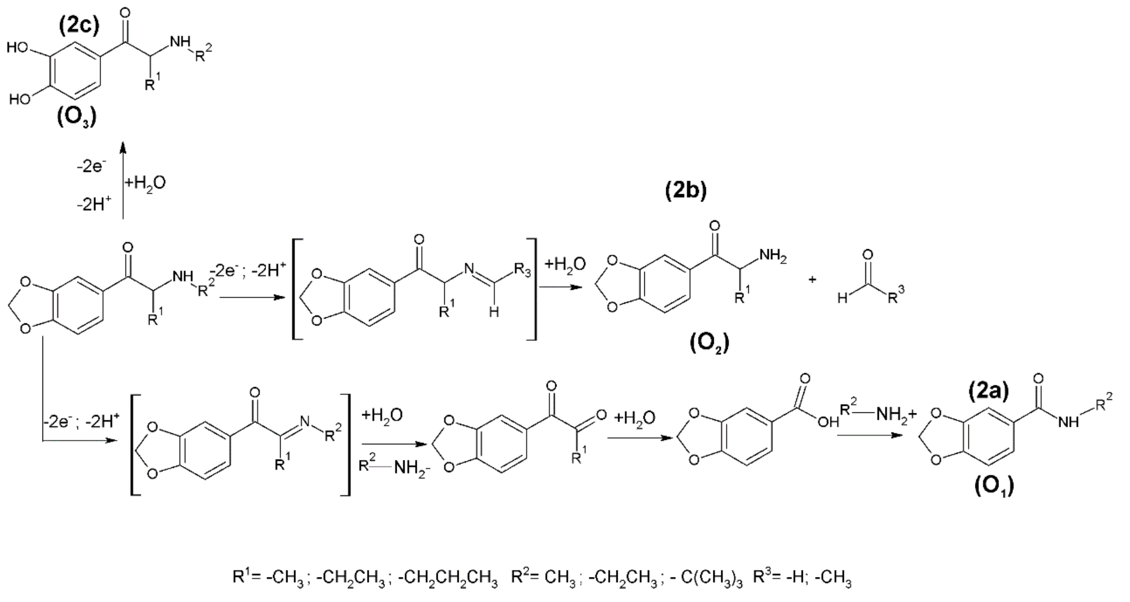

Figure S16. Proposed oxidation mechanism for SCs derivatives in the G2 group.

As previously reported,[5] the product 2a (Figure S16) is associated with the formation of a small amount of benzamide. Therefore, it is assumed that product 2a corresponds to the O_1 process, since this process exhibited a low peak current (Fig. S13B-C). Structure 2b (Figure S16) is linked to the O_2 anodic process, representing the oxidation of the secondary amine, similar to the O_1 process observed in G1 group cathinones. Electrolysis of methylone solutions at +1.27 V in basic medium led to the formation of 3,4-dihydroxymethcathinone,[5] which aligns with the O_3 process of G2 group SCs, corresponding to product 2c (Figure S16).

The origin of the O_1 and O_2 processes observed in the electrochemical profile of group G5 analogs was studied by Schram *et al.*[5] using electrolyzed solutions of the cathinone 4-chloro-alpha-pyrrolidinovalerophenone (Cl-PVP). Like MPHP, alpha-PVP, and TH-PVP, Cl-PVP and the G1 group analogs do not have a 3,4-methylenedioxy ring. Thereby, the O_1 and O_2 processes involve oxidations of the pyrrolidine ring.[1] Figure S17 presents a proposed generic mechanism for G5 group SCs, based on the electrolyzed products identified by Schram *et al.*[5]

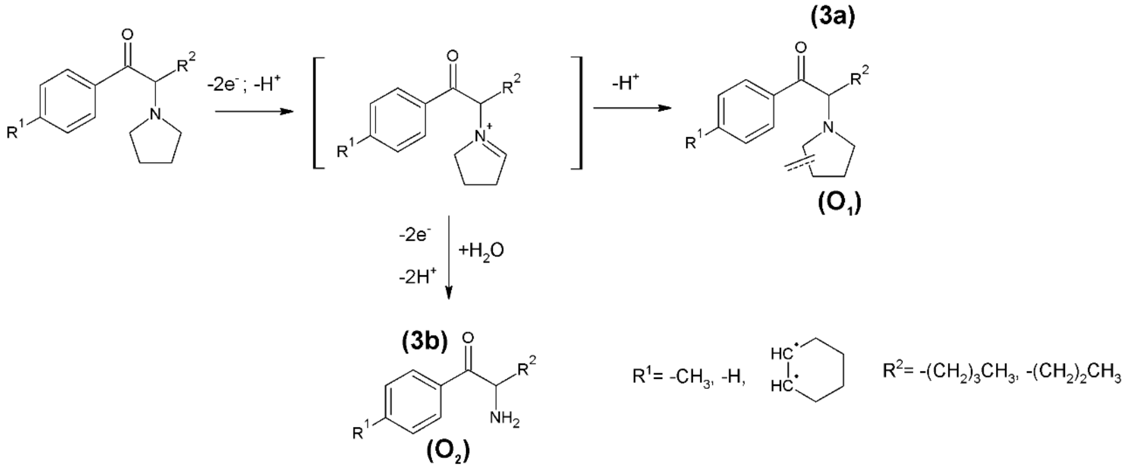

Figure S17. Proposed oxidation mechanism for SCs derivatives in the G5 group.

According to evidence presented by Schram *et al.*,[5] the O_1 process corresponds to the formation of product 3a, which was observed in the electrolyzed solutions around + 0.7 V. Product 3a forms by the abstraction of electrons from the non-bonding nitrogen pair, followed by the formation of a double bond (Figure S17). The O_2 process at around +1.0 V was confirmed by HPLC-MS, corresponding to the oxidation of the pyrrolidine ring to a primary amine (3b).

The mechanisms of other SC groups presented in this work (G3, G4, and G6) were not explored by Schram *et al.*[5] However, due to structural similarities and comparable electrochemical profiles, the anodic processes of these groups can be proposed based on analogies to the G1, G2, and G5 groups.

Dibutylone, a G3 group cathinone, differs from G2 structures by the presence of a tertiary amine in its alkyl chain. As shown in Figure S14A, the DPVs of dibutylone indicate that the O_1, O_3, and O_4 processes correspond to the O_1, O_2, and O_3 processes of G2 SCs (Figure S13B-C). According to the generic mechanism proposed for G2 group cathinones (Figure S16), for dibutylone, the O_1 process corresponds to benzamide formation, O_2 and O_3 are related to the tertiary amine oxidation, and O_4 results from the opening of the 3,4-methylenedioxy ring.

Structurally, MDPV and MDPHP combine features of G2 (3,4-methylenedioxy ring) and G3 (pyrrolidine ring), accounting for their anodic processes. The oxidation mechanisms of MDPV and MDPHP are thus a combination of G2 and G3 mechanisms. As in G2 (Figure S16), the O_1 process of MDPV and MDPH corresponds to benzamide formation, and the O_5 peak indicates the formation of a dihydroxy structure from the

opening of the 3,4-methylenedioxy ring. As discussed for MPHP, α-PVP, and TH-PVP, the O_2, O_3, and O_4 processes of MDPV and MDPH are attributed to pyrrolidine ring oxidation.

SC α-PVT (G6) is structurally distinct from other cathinones due to its thiophene ring. However, its alkyl chain includes a pyrrolidine ring, similar to G3 and G4 cathinones. Therefore, the anodic processes O_3 and O_4 in the α-PVT voltammogram (Figure S14D) have the same peak potential as the anodic processes O_2 and O_3 in G4, and O_1 and O_2 in G5, and are similarly attributed to pyrrolidine ring oxidation (Figure S17).

Regarding the mechanisms underlying the cathodic processes in the six SC groups, only the R_1 process has a reported mechanism.[7] One of the most common organic transformations is the reduction of ketones to alcohols, followed by dehydration to form an alkene.[8] Thus, the proposed mechanisms for the R_1 and R_2 reductions are based on these transformations, where the conversion of alcohols to alkenes requires an acidic environment and high temperatures.[9] In this work, the acidic environment is provided by the cathodic pretreatment on the BBDE surface. The applied potential supplies the energy required for the two-step electrochemical conversion of the ketone to an alkene. Figure S18 illustrates the proposed mechanism for the formation of the R_1 and R_2 processes, serving as a generic proposal for all SCs investigated.

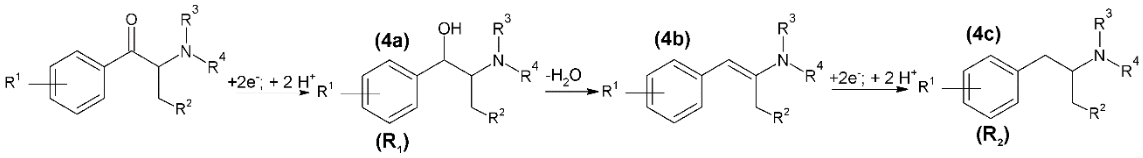

Figure S18. Proposed reduction mechanism for all investigated SCs.

The R_1 cathodic process of SCs produces a well-defined product, which, according to studies on the biological metabolism of mephedrone, corresponds to the reduction of the β-keto moiety of SCs to its corresponding alcohol (4a).[7] The R2 process involves the conversion of the alcohol formed in R1 (4a) into an alkene (4b) through a dehydration reaction via an elimination mechanism,[9] followed by hydrogenation. Although the alkene formed in 4b is more stable due to the presence of an additional conjugated double bond, which increases charge distribution across the molecule, it still undergoes hydrogenation, leading to the formation of the corresponding alkane (4c).

Table S2. Intra-day and inter-day repeatability (N = 5) for the R_1 process of 100 µmol L^{-1} SC standards.

**SCs**	**Intra-day repeatability**		**Inter-day repeatability**	
	***Epc_1* RSDs (%)**	***Ipc_1* RSDs (%)**	***Epc_1* RSDs (%)**	***Ipc_1* RSDs (%)**
4-MMC	0.32	3.2	0.33	4.1
Ethcathinone	0.43	4.5	0.54	4.8
4-MPD	0.01	4.3	0.01	8.8
bk-MDMA	0.01	3.5	0.01	3.2
Bk-MDEA	0.30	1.6	0.33	3.4
bk-EBDB	0.33	2.5	0.66	2.0
Ephylone	0.33	0.7	0.32	2.0
MDPT	0.33	3.8	0.02	2.9
bk-DMBDB	0.42	4.6	0.01	3.8
MDPV	0.37	5.1	0.34	11.1
MDPHP	0.01	3.2	0.34	3.3
MPHP	0.01	3.4	0.01	3.1
α-PVP	0.35	7.2	0.43	8.5
TH-PVP	0.67	12.8	0.60	12.6
α-PVT	0.01	5.0	0.44	9.0

*Experimental conditions: 0.1 mol L^{-1} BR buffer at pH 8.0, BDDE, and DPV technique (80 mV amplitude, 10 mV step potential, 50 ms modulation time, and 0.1 s time interval).

Pulsed voltammetric technique optimization

Since synthetic cathinones do not exhibit reversible processes, both DPV and SWV were considered for detection. To address this, we optimized both techniques using MPHP as a model analyte. Under optimal conditions, analytical curves were obtained for MPHP with both techniques, as shown in Figure S19. Both DPV and SWV exhibited similar sensitivities and limits of detection, with values of 0.048 ± 0.001 (μA / μmol L^{-1}; LOD= 3.8 μmol L^{-1}) for DPV (Fig. S19A) and 0.052 ± 0.002 (μA / μmol L^{-1}; LOD= 3.7 μmol L^{-1}) for SWV (Fig. S19C). However, DPV demonstrated better stability across different concentrations, with relative standard deviations (RSDs) < 5 % in the calibration curves for triplicate measurements at each concentration, as reflected in the error bars (Fig. S19A). Additionally, DPV provided a wider linear working range. Based on these results, DPV was selected as the preferred technique for SC detection in the proposed method.

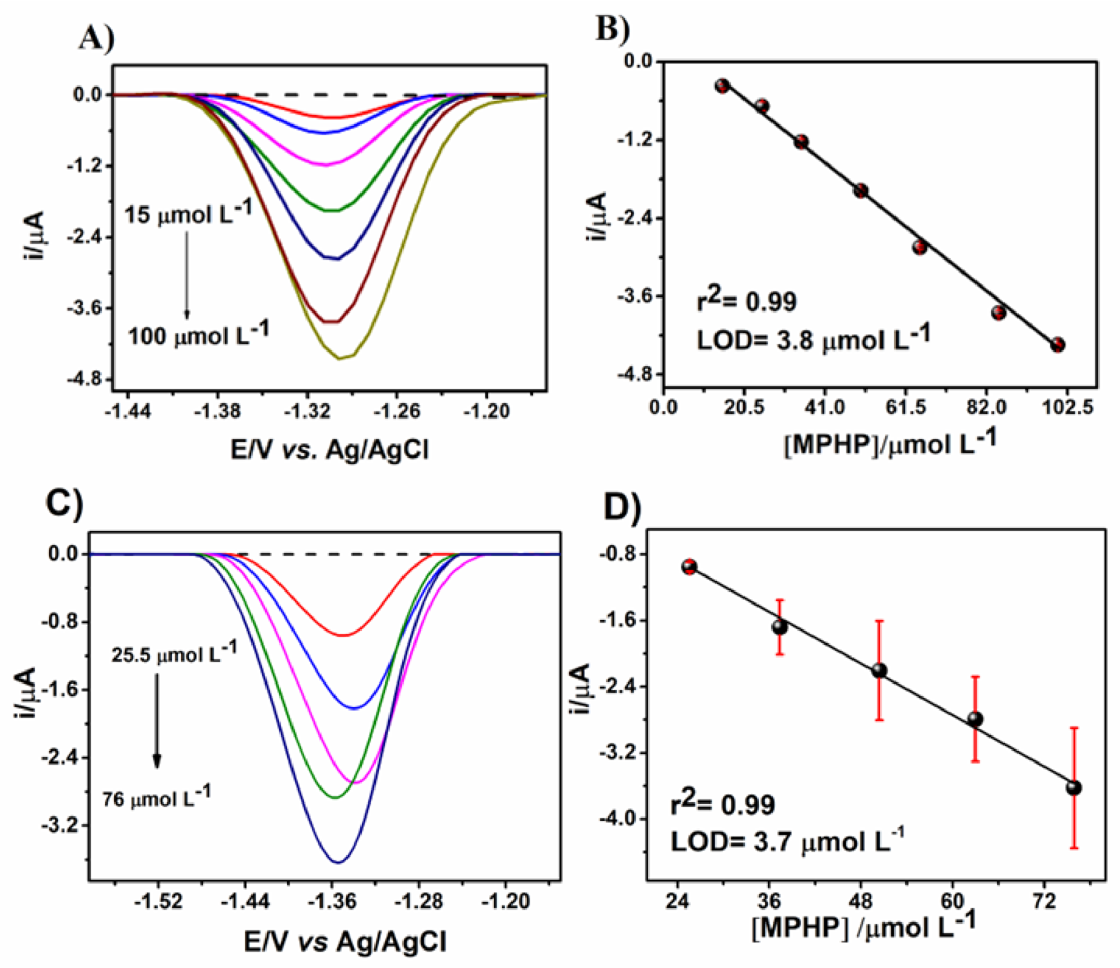

Figure S19. (A) DPVs on BDDE of 15 – 100 µmol L^{-1} MPHP and **(B)** its respective calibration curve: I (µA) = - 0.42 (± 0.06) - 0.048 (± 0.001) [MPHP] (µA / µmol L^{-1}). **(C)** SWVs on BDDE of 25.5 – 76 µmol L^{-1} MPHP obtained by cathodic scans and (**D**) its respective calibration curve: I (µA) = - 0.32 (± 0.06) - 0.052 (± 0.002) [MPHP] (µA / µmol L^{-1}). Experimental conditions: 0.1 mol L^{-1} BR buffer at pH 8.0 was used as supporting electrolyte for both techniques, with current peaks obtained from R_1 using cathodic scans. Optimized parameters for DPV: 80 mV amplitude, 10 mV step potential, 50 ms modulation time, and 0.1 s time interval; for SWV: 50 mV amplitude, 20 HZ Frequency and 5 mV step potential.

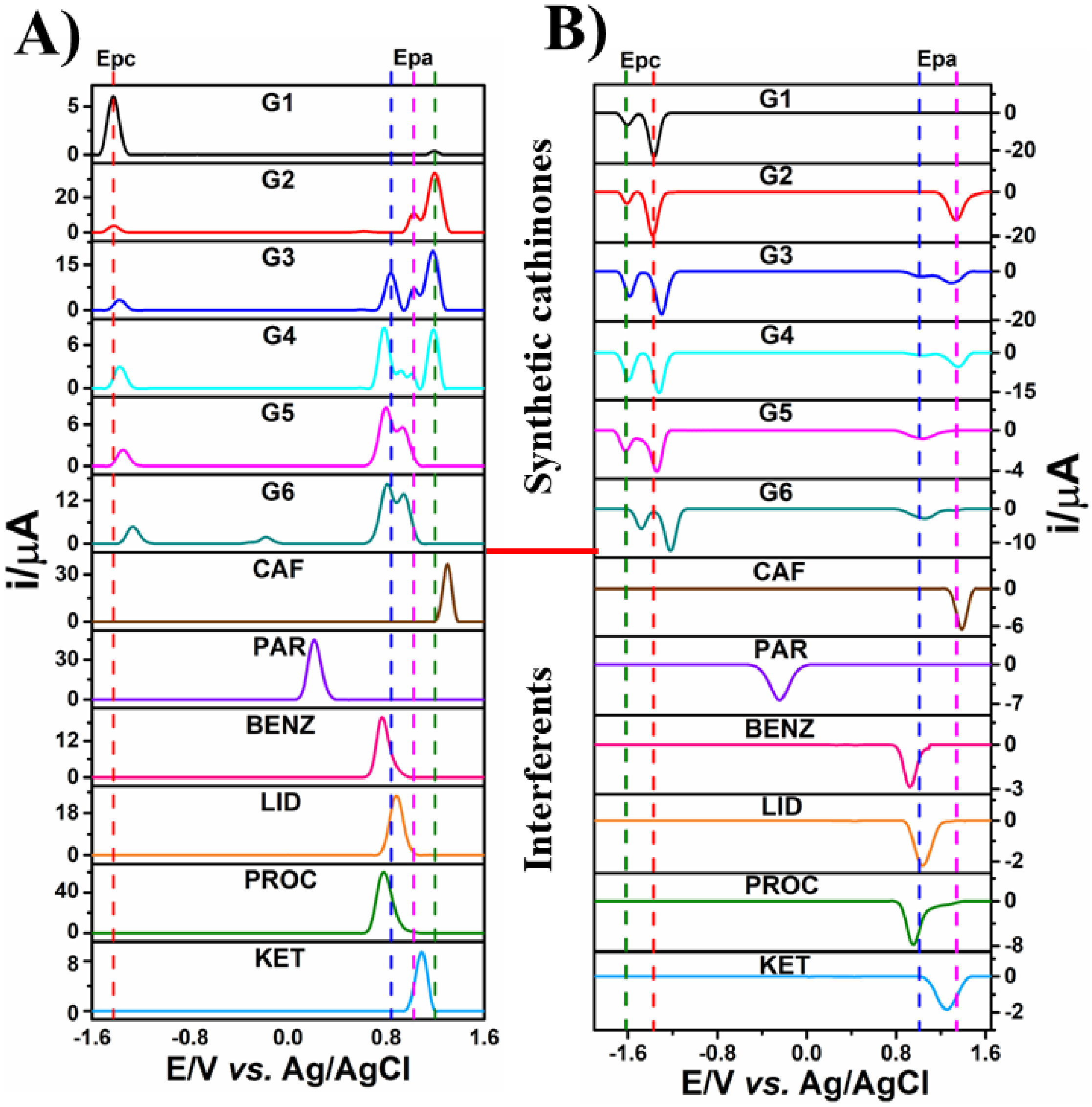

Figure S20. DPVs on BDDE using anodic (A) and cathodic (B) scans in the presence of SCs representatives from groups G1 to G6 and interferents such as caffeine, paracetamol, and anesthetic medications.

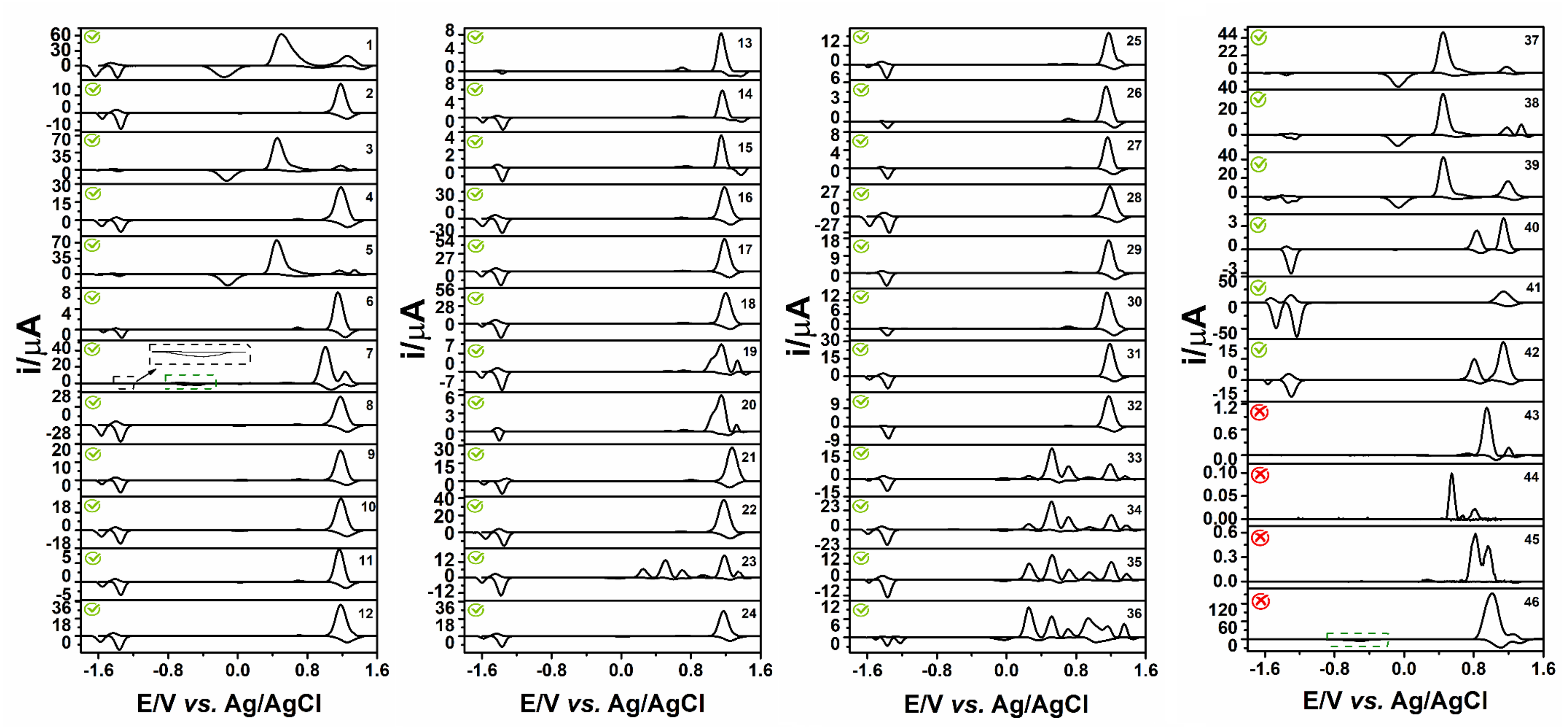

Figure S21. DPVs using anodic and cathodic scans with baseline correction, obtained from screening SCs in real samples diluted 800x in 0.1 mol L^{-1} BR buffer at pH 8.0. Experimental conditions: 80 mV amplitude, 10 mV step potential, 50 ms modulation time, and 0.1 s time interval; anodic sweep potential window from - 1.9 to + 2.0 V and cathodic sweep from + 2.0 to - 1.9 V.

A)

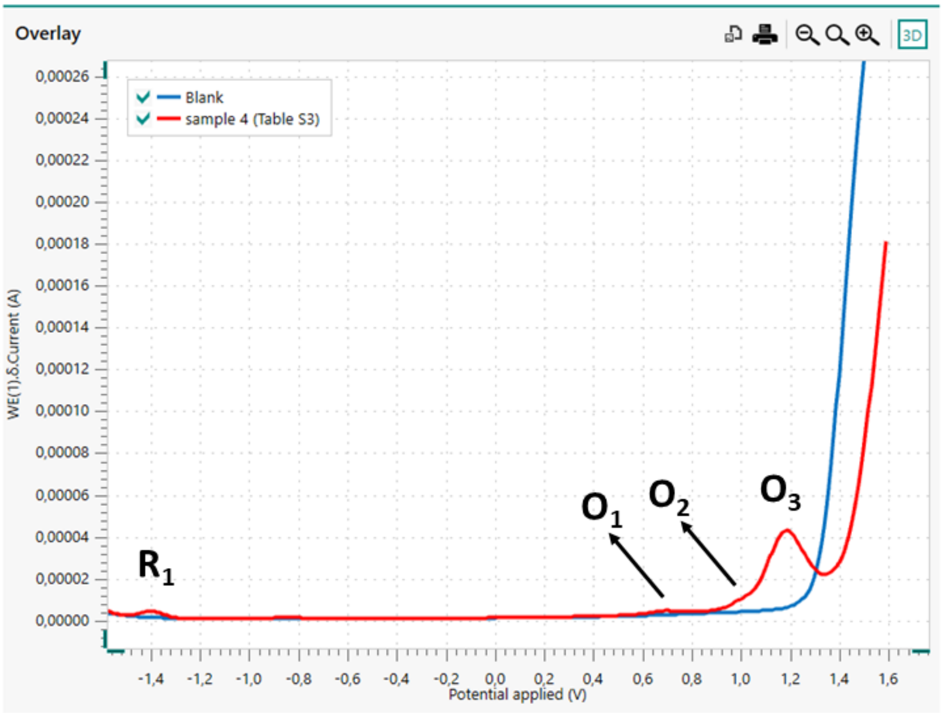

B)

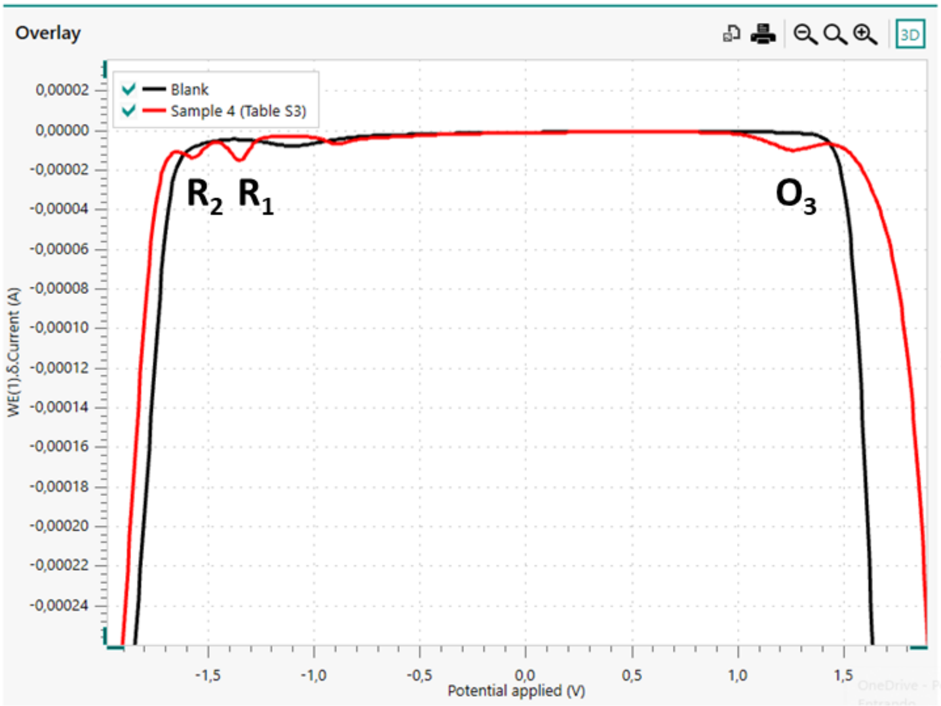

Figure S22. DPVs using anodic and cathodic scans without baseline correction, obtained from real sample 4 diluted 800x in 0.1 mol L^{-1} BR buffer at pH 8.0. The experimental conditions were the same as in Figure S21.

Table S3. Comparison of results from the DPV-BDDE screening method for SCs with LC-MS results for seized samples.

Samples	DPV-BDDE method	SC group based on DPV-BDDE results	SC group based on LC-MS results
1	+	G2	Ephylone (G2)
2	+	G2	Ephylone (G2)
3	+	G2+CAF	Ephylone (G2) /PAR/CAF
4	+	G2	Ephylone(G2) /MDMA
5	+	G2	Ephylone (G2)
6	+	G2	Ephylone (G2)
7	+	G2+MDMA/MDEA	Ephylone(G2) /MDMA/CAF
8	+	G2	Ephylone(G2) /4-MPD (G1)
9	+	G2	Ethylone (G2) / CAF/KET
10	+	G2	Ethylone (G2) /CAF/KET
11	+	G2	Ethylone (G2)
12	+	G2	Ethylone (G2)
13	+	G2	MDPT (G2)
14	+	G2	MDPT (G2)
15	+	G2	MDPT (G2)
16	+	G2	MDPT (G2)
17	+	G2	MDPT (G2)
18	+	G2	MDPT (G2)
19	+	G2+CAF	BMDP (G2)/CAF
20	+	G2+CAF	BMDP (G2)/CAF
21	+	G2	Eutylone (G2)
22	+	G2	Eutylone (G2)
23	+	G2+CAF	Eutylone (G2)/CAF
24	+	G2	Eutylone (G2)
25	+	G2+CAF	Eutylone (G2)
26	+	G2	Eutylone (G2) /CAF
27	+	G2	Eutylone (G2) /CAF
28	+	G2	4-MPD (G1) /Ephylone (G2)
29	+	G2+CAF	Eutylone (G2) /CAF
30	+	G2+CAF	Eutylone (G2) /CAF/KET
31	+	G2	Ethylone (G2)
32	+	G2	Eutylone (G2)
33	+	G2+CAF	Eutylone (G2) /CAF
34	+	G2+CAF	Eutylone (G2) /CAF
35	+	G2+CAF	Eutylone (G2) /CAF
36	+	G2+CAF	BMDP (G2) / CAF
37	+	G2	Ephylone (G2)
38	+	G2+CAF	Ephylone (G2) /CAF/ PAR/DIC/CAR
39	+	G2+CAF	Ephylone (G2) /CAF/ PAR/DIC/CAR
40	+	G3	Dipentylone(G3)
41	+	G1	Bupropion (G1)
42	+	G3	Dipentylone (G3)
43	-	-	-
44	-	-	-
45	-	-	-
46	-	MDMA/MDEA	MDMA

BMDP: 3,4-methylenedioxy-N-benzylcathinone, DIC: diclofenac, CAR: carisoprodol.

REFERENCES

(1) B. Oliveira, S. C.; Oliveira-Brett, A. M. Voltammetric and Electrochemical Impedance Spectroscopy Characterization of a Cathodic and Anodic Pre-Treated Boron Doped Diamond Electrode. *Electrochim Acta* **2010**, *55* (15), 4599–4605. https://doi.org/10.1016/J.ELECTACTA.2010.03.016.

(2) Muzyka, K.; Sun, J.; Fereja, T. H.; Lan, Y.; Zhang, W.; Xu, G. Boron-Doped Diamond: Current Progress and Challenges in View of Electroanalytical Applications. *Analytical Methods*. Royal Society of Chemistry January 28, 2019, pp 397–414. https://doi.org/10.1039/c8ay02197j.

(3) Pimentel, D. M.; Arantes, L. C.; Santos, L. M.; Souza, K. A. O.; Verly, R. M.; Barbosa, S. L.; dos Santos, W. T. P. Rapid and Simple Voltammetric Screening Method for Lysergic Acid Diethylamide (LSD) Detection in Seized Samples Using a Boron-Doped Diamond Electrode. *Sens Actuators B Chem* **2021**, *344*, 130229. https://doi.org/10.1016/J.SNB.2021.130229.

(4) Scheel, G. L.; de Oliveira, F. M.; de Oliveira, L. L. G.; Medeiros, R. A.; Nascentes, C. C.; Tarley, C. R. T. Feasibility Study of Ethylone Determination in Seized Samples Using Boron-Doped Diamond Electrode Associated with Solid Phase Extraction. *Sens Actuators B Chem* **2018**, *259*, 1113–1122. https://doi.org/10.1016/J.SNB.2017.12.129.

(5) Schram, J.; Parrilla, M.; Sleegers, N.; Van Durme, F.; van den Berg, J.; van Nuijs, A. L. N.; De Wael, K. Electrochemical Profiling and Liquid Chromatography–Mass Spectrometry Characterization of Synthetic Cathinones: From Methodology to Detection in Forensic Samples. *Drug Test Anal* **2021**, *13* (7), 1282–1294. https://doi.org/10.1002/dta.3018.

(6) Garrido, E. M. P. J.; Garrido, J. M. P. J.; Milhazes, N.; Borges, F.; Oliveira-Brett, A. M. Electrochemical Oxidation of Amphetamine-like Drugs and Application to Electroanalysis of Ecstasy in Human Serum. *Bioelectrochemistry* **2010**, *79* (1), 77–83. https://doi.org/10.1016/J.BIOELECHEM.2009.12.002.

(7) Pedersen, A. J.; Reitzel, L. A.; Johansen, S. S.; Linnet, K. In Vitro Metabolism Studies on Mephedrone and Analysis of Forensic Cases. *Drug Test Anal* **2013**, *5* (6), 430–438. https://doi.org/10.1002/DTA.1369.

(8) Clark, A. J.; Guy, C. S. Reduction of Ketones to Alkenes. In *Comprehensive Organic Synthesis: Second Edition*; Elsevier Ltd., 2014; Vol. 8, pp 1143–1163. https://doi.org/10.1016/B978-0-08-097742-3.00833-8.

(9) Biggs, R. A.; Ogilvie, W. W. Eliminations to Form Alkenes, Allenes, and Alkynes and Related Reactions. In *Comprehensive Organic Synthesis: Second Edition*; Elsevier Ltd., 2014; Vol. 6, pp 802–841. https://doi.org/10.1016/B978-0-08-097742-3.00627-3.
